# *miR-146a* Enhances the Oncogenicity of Oral Carcinoma by Concomitant Targeting of the IRAK1, TRAF6 and NUMB Genes

**DOI:** 10.1371/journal.pone.0079926

**Published:** 2013-11-26

**Authors:** Pei-Shi Hung, Chung-Ji Liu, Chung-Shan Chou, Shou-Yen Kao, Cheng-Chieh Yang, Kuo-Wei Chang, Ting-Hui Chiu, Shu-Chun Lin

**Affiliations:** 1 Department of Surgery National Yang-Ming University Hospital, Yi-Lan, Taiwan; 2 Department of Medical Research, National Yang-Ming University Hospital, Yi-Lan, Taiwan; 3 Institute of Oral Biology, National Yang-Ming University, Taipei, Taiwan; 4 Department of Dentistry, National Yang-Ming University, Taipei, Taiwan; 5 Department of Oral and Maxillofacial Surgery, Taipei Mackay Memorial Hospital, Taipei, Taiwan; 6 Department of Stomatology, Taipei Veterans General Hospital, Taipei, Taiwan; Virginia Commonwealth University, United States of America

## Abstract

MicroRNAs are short non-coding RNAs that regulate gene expression and are crucial to tumorigenesis. Oral squamous cell carcinoma (OSCC) is a prevalent malignancy worldwide. Up-regulation of *miR-146* has been identified in OSCC tissues. However, the roles of *miR-146* in carcinogenesis are controversial as it is suppressive in many other malignancies. The present study investigated the pathogenic implications of *miR-146a* in oral carcinogenesis. Microdissected OSCC exhibits higher levels of *miR-146a* expression than matched adjacent mucosal cells. The plasma *miR-146a* levels of patients are significantly higher than those of control subjects; these levels decrease drastically after tumor resection. *miR-146a* levels in tumors and in patients’ plasma can be used to classify OSCC and non-disease status (sensitivity: >0.72). Exogenous *miR-146a* expression is significantly increased *in vitro* oncogenic phenotypes as well as during xenograft tumorigenesis and OSCC metastasis. The plasma *miR-146a* levels of these mice parallel the xenograft tumor burdens of the mice. A *miR-146a* blocker abrogates the growth of xenograft tumors. *miR-146a* oncogenic activity is associated with down-regulation of IRAK1, TRAF6 and NUMB expression. Furthermore, *miR-146a* directly targets the 3′UTR of NUMB and a region within the NUMB coding sequence when suppressing NUMB expression. Exogenous NUMB expression attenuates OSCC oncogenicity. Double knockdown of IRAK1 and TRAF6, and of TRAF6 and NUMB, enhance the oncogenic phenotypes of OSCC cells. Oncogenic enhancement modulated by *miR-146a* expression is attenuated by exogenous IRAK1 or NUMB expression. This study shows that *miR-146a* expression contributes to oral carcinogenesis by targeting the IRAK1, TRAF6 and NUMB genes.

## Introduction

MicroRNAs (miRNAs) are small non-protein-coding RNA molecules of 20–22 nucleotides that negatively regulate the expression of target genes. Many miRNAs are involved in carcinogenesis, inflammation and other pathological process [1]. Head and neck squamous cell carcinoma (HNSCC), including oral squamous cell carcinoma (OSCC), is one of the most prevalent malignancies worldwide [2]–[4]. We have previously identified an association between alterations in miRNA expression and the OSCC progression [2], [3].

NFκB is an important gene regulator that plays central roles in the immune response, inflammation, stress responses, reactions to drugs and apoptosis [5]–[7]. This protein seems to have dual functions in tumorigenesis, where it can act either as a tumor promoting gene or as a tumor suppressor gene [7]. *miR-146a* is up-regulated by the NFκB signal pathway, which is activated by Toll-like receptor (TLR), tumor necrosis factor α receptor (TNFR), interleukine 1β receptor (IL1R), receptor activator of NFκB (RANK) and other upstream elements. On the other hand, IL-1 receptor–associated kinase 1 (IRAK1) and TNF receptor-associated factor 6 (TRAF6), critical adapter molecules in this pathway, have been shown to be target genes of *miR-146a* [8]. *miR-146a* has been shown to be a negative feedback regulator of the TLR signal pathway, which is involved in inflammatory pathogenesis [9]. The NFκB-*miR-146a* regulatory loop has been suggested to be an important causal link between inflammation and carcinogenesis. NFκB is able to affect the oncogenic potential of various malignancies including HNSCC [10], [11]. The constitutive activation of the TNF-TNFR1-TRAF2 cascade, rather than the RANKL-RANK-TRAF6 or IL-1β-IL1R-IRAK cascades, has been previously reported to underlie IKK stabilization and constitutive NFκB activation in HNSCC cells [10]. High expression of TLR4 in HNSCC tumor cells in response to inflammatory stimuli has been found to be associated with IRAK4 up-regulation, AKT phosphorylation, NFκB activation and the increased proliferation of tumor cells [11]. *miR-146a* has also been shown to be an interferon regulator by targeting IRF5 and STAT1 [12]; a TGFβ pathway regulator by targeting Smad4 [13], [14]; and a regulator of ROCK-1, EGFR, L1-CAM and MCP-2 in various types of cells [15]–[20].

NUMB is a highly conserved protein that exhibits high structural complexity and has multiple alternative spliced forms; these drive a diverse range of physiological or pathogenic regulatory system [21]. It was originally found to antagonize NOTCH during the modulation of neural differentiation [22]. NUMB also functions as a tumor suppressor by abrogating Gli and/or NOTCH in glioblastoma and breast cancer [23], [24]. In addition, NUMB isoform 4, one of the predominant NUMB isoforms, decreases both NOTCH and EGFR expression in glioblastoma cells [25]. NUMB also stabilizes p53 by inactivating mdmd2 [26]. A recent report has indicated that TNFα-induced FoxA2 phosphorylation is responsible for the down-regulation of NUMB, which then facilitates breast cancer tumorigenesis by enhancing NOTCH [27]. NUMB has been shown to be targeted by *miR-146a* in C2C12 myogenic cells [16]; nevertheless, the role of NUMB and the effect of NFκB/*miR-146a* regulation on NUMB during epithelial pathogenesis remains obscure.

Up-regulation of *miR-146* has been found in HNSCC, squamous cell carcinoma (SCC) of the cervix, SCC of the lung, melanoma, gastric carcinoma and thyroid carcinoma [13], [14], [28]–[34]. High *miR-146b* expression has been shown to define a poor prognosis in patients with SCC of the lung [34]. Exogenous *miR-146a* expression is known to increase the proliferation of several kinds of cells [28], [30], [35]. This contrasts with the fact that *miR-146a* is suppressive in breast, gastric, prostatic and pancreatic carcinomas [15], [20], [36]–[38], and that down-regulation of *miR-146a* is associated with the pathogenesis of hematopoietic malignancies [39]. Taking the above findings as a whole, *miR-146a* seems to play a wide variety of roles in the regulation of different phenotypes and is able to target a wide range of different genes in various cellular microenvironments; this has led to controversy as to its role in carcinogenesis. The TNFα and TLR signal cascades, which are linked to *miR-146a* expression, are important triggers for HNSCC tumorigenesis [10], [11]. Our previous study identified that there is higher expression of *miR-146a* in OSCC tissues carrying the C-variant functional polymorphism [30]. Nevertheless, the extent to which changes in *miR-146a* expression impacts on OSCC cells remains largely unclear. This study addressed the oncogenic roles of *miR-146a* in OSCC and identified that IRAK1, TRAF6 and NUMB are targets of *miR-146a* in OSCC cells.

## Materials and Methods

### OSCC Tissue and Plasma Samples

Surgical tissue specimens and blood samples from OSCC patients were collected after obtaining written informed consent and this study was approved by The Institutional Review Board in Mackay Memorial Hospital (IRB approval no. 09MMHIS146). The surgical specimens included primary tumors along with paired non-cancerous matched tissues (NCMT) (Table S1). The tumors underwent TNM classification according to the American Joint Committee on Cancer (AJCC) system. Microdissection was performed to retrieve pure epithelial components from the tissue sections and this was carried out according to established protocols [3]. Whole blood (5 ml) was collected in a heparin-coated tube from the patients [40]. Twelve age-matched male subjects served as controls.

### qRT-PCR Analysis

Total RNA was isolated from the microdissected tissue or plasma samples and these samples was then used as input to assay *miR-146a* expression using TaqMan MicroRNA Assays kits (Applied Biosystems, Foster City, CA) according to the manufacturer’s instructions. PCR reactions were carried out on a Quantica® real time nucleic acid detection system (Techne Inc., Burlington, NJ) using *RNU6B* (for the tissue analysis) and *miR-16* (for the plasma) as the internal controls [2], [40]. *NUMB* mRNA expression was assayed using TaqMan Gene Expression Assay systems (Applied Biosystems). The comparative threshold cycle (Ct) method was used to measure relative changes in expression [2], [3]. 2^ΔΔCt^ represents the fold change in expression. A negative control without a template was run in parallel to assess the overall specificity of the reaction.

### Cell Culture and Reagents

The OSCC cell lines FaDu, HSC3, OECM-1 and SAS cells, normal human oral keratinocytes (NHOKs), as well as 293FT cells, were cultured as previously described [2], [41]–[43]. Chemically modified pre-*miR-146a* mimic and it scramble control (Scr), as well as *miR-146a* locked nucleic acid (*miR-146a* LNA) blocker together with its scramble control (Scr LNA) were purchased from Applied Biosystems. The small interference oligonucleotides siIRAK1, siTRAF6 and siNUMB, which were used to knock down gene expression, as well as the control oligonucleotide (siControl), were purchased from Santa Cruz Biotech (Santa Cruz, CA). TransFectin™ (Bio-Rad, Hercules, CA) lipid reagent was used as the transfection reagent. TNFα was purchased from Sigma-Aldrich (St Louise, MO). Unless specified otherwise, all other reagents were purchased from Sigma-Aldrich.

### Plasmid Construction and Generation of Lentiviruses

The pri-pre-*mir-146a* sequence was amplified from genomic DNA, digested by restriction enzymes and cloned into a lentivirus vector harboring a green fluorescence (GFP) tag [2]. The plasmid was co-transfected with helper plasmids into 293FT cells to produce lentiviruses. The OSCC cells were infected with the lentiviruses carrying *miR-146a* or GFP. The infected cells were subjected to fluorescence-activated cell sorting using a FACSCalibur apparatus (Becton Dickinson, Franklin Lakes, NJ) in order to isolate fluorescent cell subclones. The expression level of *miR-146a* in the cell subclones was determined by qRT-PCR analysis.

p65 plasmid was used for the exogenous expression of the NFκB p65 subunit [44]. The pCMVSPORT6-IRAK1 plasmid clone (NIH_MGC_72) obtained from Mammalian Gene Collection (MGC). This plasmid contains the IRAK1 coding sequence (CDS) and its 3′UTR, was used for exogenous IRAK1 expression. Empty vector was used as vector alone (VA) controls.

Antisense sequences complementary to the mature *miR-146a* sequences were cloned into the 3′-end of the *lacZ* coding sequence of the pCMV-LacZ plasmid, which was then used to measure *miR-146a* activity [2], [3]. The 3′UTRs of SIAH2 and ST7L, which contain predicted *miR-146a* target sites, were also cloned into the pCMV-LacZ reporter plasmid. The reporter constructs were designated SIAH2-R and ST7L-R. Co-transfection of the reporters together with the pCMV-Luc plasmid into cells was then performed. After normalization against luciferase activity, β-galactosidase activity should be repressed if *miR-146a* targeting activity is present in the cells.

Two types of reporters were generated to detect whether *miR-146a* targets the 3′UTR of the NUMB gene. The NUMB-3′UTR-WtR(B) reporter was generated by cloning the 3′UTR of the wild-type NUMB sequence into pCMV-LacZ. The NUMB-3′UTR-MutR(B) reporter construct was generated from NUMB-3′UTR-WtR(B) by replacing the target sequence AGUUCUCA with AACUAGUA. Similarly, the NUMB-3′UTR-WtR(G) reporter was generated by cloning the wild-type 3′UTR sequence of NUMB into pCMV-GFP. The NUMB-3′UTR-MutR(G) reporter was then generated from NUMB-3′UTR-WtR(G) by in vitro mutagenesis. For both types of reporters, co-transfection with pCMV-Luc plasmid into cells was carried out as a transfection control to allow normalization of β-galactosidase activity or GFP expression. Luciferase assays were performed using a luciferase detection kit (Promega, Madison, WI).

For exogenous NUMB expression, the wild-type CDS of NUMB (isoform 4) and wild-type full-length NUMB cDNA, including the CDS and 3′UTR, were cloned into pBabe-puro vector to produce constructs that were designated NUMB CDS-Wt and NUMB CDS+3′UTR, respectively. As six nucleotides in the CDS of NUMB were predicted to contain a potential target site for *miR-146a*, a mutant construct designated NUMB CDS-Mut was generated by replacing ACACCTGAGGACCCCTTCTCA in NUMB coding sequence with ACACCAGAGGACCCCTTTAGC ; this change does not affect the amino acid sequence of NUMB. Co-transfection of pCMV-GFP was used as a transfection control.

The short hairpin RNA (shRNA) vectors for the knockdown of IRAK1, TRAF6 and NUMB (Table S2) were obtained from the RNA interference consortium (Academia Sinica, Taipei, Taiwan). shLuc was the control [45]. Lentiviruses were generated by co-transfecting 293T cells with lentiviral vectors and packaging plasmids. The infected SAS cells were selected with puromycin to achieve stable cell subclones [45].

### NFκB Activity Assay

A luciferase reporter vector and a NFκB reporter plasmid derived from this plasmid, which contains a NFκB response element insert, were co-transfected with a pCMV-LacZ plasmid into cells [44]. NFκB transcription activity in the cells was determined after normalization against β-galactosidase activity and luciferase activity in vector alone transfected cells.

### Western Blot Analysis

Western blot analysis followed previously described protocols [3]. The providers and dilution of the primary antibodies that were used are presented in Table S3. A total of 40 µg of cell lysate or protein that had been isolated from each tissue specimen were loaded for analysis.

### Cell Proliferation

The viability of cells was measured by Trypan blue exclusion assay. The ratios of viable cells at different time points relative to the total number of cells seeded were plotted and analyzed statistically.

### Invasion Assay

For the invasion assay, cells were grown in serum-free media on a Transwell (Corning, Acton, MA) with a porous transparent polyethylene terephthalate membrane having a pore diameter of 12 µm; this membrane had been coated with 1∶4 Matrigel basement membrane matrix (BD Biosciences, San Jose, CA). Cell growth was arrested by treatment with 1 µM hydroxyurea after 48 h and then the cells on upper surface of the membrane were wiped off. Finally, the cells attached to the lower surface were counted by fluorescence microscopy following staining with Hoechst 33258.

### Anchorage-independent Growth (AIG)

Cells were suspended in 1.3% methylcellulose in culture media, plated on a layer of 0.9% agarose in culture media in six-well culture plates and then cultured at 37°C. After 7 to 10 days, the number of colonies present in five fields per well, giving a total of 15 fields in triplicate experiments, were counted [3].

### Tumorigenesis, Metastasis and Experimental Therapy

For the induction of subcutaneous tumor or xenografic tumor growth on lateral tongue, 2.5×10^5^ SAS, 2.5×10^5^ FaDu cells or 1×10^6^ OECM-1 cells were injected subcutaneously into the flank or the lateral tongue of 6–8-week-old athymic mice. Tumor volumes were calculated using the formula = *0.5*×*a*×*b* ^2^, where *a* and *b* are the long and short diameters of the tumors, respectively [3]. The mice were sacrificed at the end point and the tumors were resected. The tumors were then weighed, photographed and subjected to histopathological evaluation.

For the induction of primary orthotopic tumor, neck nodal metastasis and potential distal metastasis, 5×10^5^ cells from various SAS cell subclones were injected into the central portion of tongue of 6–8-week-old Non-Obese Diabetic/Severe Combined Immunodeficiency (NOD/SCID) mice. The mice were sacrificed at the time points optimized in pilot tests. Indian ink was injected into tongue tissue to facilitate node dissection. In addition, the head and neck region was photographed under an illumination device (LT-9500, Illumatool, TLS) to visualize the positive nodes. Each whole tongue was resected and subjected to histopathological evaluation. Neck tissues were removed by radical dissection of tissue exhibiting green fluorescence, black staining or a firm-feel, together with any suspicious masses; these were then embedded for histopathological examination. Finally, a complete autopsy was performed to allow the internal organs to undergo histopathological examination. Tumor volumes were calculated using the formula = *0.5*×*a*×*b* ^2^, where *a* and *b* are the long and short diameters of the tumors measured under microscopy, respectively. In addition, around 0.2 ml of blood was sampled by retro orbital puncture from mice at consecutive time points before the orthotopic inoculation, 2-week after inoculation and at the 5^th^ week of the experiments.

For distal metastasis evaluation, 5×10^5^ cells from SAS cell subclones were injected into tail vein of athymic mice. Seven weeks later, the mice were euthanized and tumor foci in lungs that showed green fluorescence were visualized by an illuminating device and photographed. The lung tissues together with any other suspicious metastatic foci were subjected to histopathological evaluation.

To test the tumorigenesis after abrogating *miR-146a*, IRAK1 and TRAF6, 0.5% atelocollagen (Koken Co., Tokyo, Japan), after it had been pepsin treated to remove antigenicity, was complexed with 2.5 µM *miR-146a* LNA or any of the other oligonucleotides in a volume of 200 µl. When the subcutaneous SAS xenografts had grown to ∼0.2 cm^3^ on athymic mice, the various complexes were individually injected subcutaneously into the periphery of tumors [3]. Tumor volumes were calculated periodically. The animal study was approved by the Institutional Animal Care and Use Committee in National Yang-Ming University (Permit Number: 981251). All procedures were performed under anesthesia, and efforts were made to minimize animal suffering during experiments by following the guidelines. In addition to scheduled endpoints, mice were sacrificed when tumors were found to be causing weakness and/or a conspicuous body weight loss (for >1/3) relative to controls.

### Statistical and Bioinformatics Analysis

The Mann-Whitney test, Wilcoxon’s test, *t*-test, Fisher’s exact test, linear regression analysis and the two-way ANOVA were used to compare differences among the clinical variants. Receiver operating characteristic (ROC) analysis and the calculation of area under curve (AUC) analysis were performed as a measure of the level of separation for the qRT-PCR analyses. Prediction algorithms were performed using the RNA22 website accessible program (http://cbcsrv.watson.ibm.com/rna22.html). A *p* value of <0.05 was considered statistically significant.

## Results

### High miR-146a Expression in OSCC

**Figure 1:**
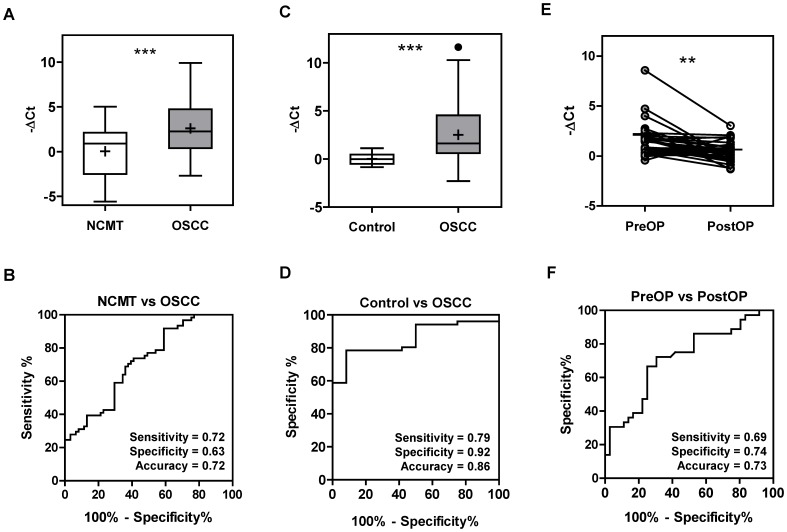
*miR-146a* expression in tissue and plasma of OSCC patients. (A) The -△Ct of *miR-146a* in OSCC tissue pairs. The findings show that there is increased *miR-146a* expression in OSCC samples relative to the corresponding NCMT samples. (C) The -△Ct of plasma *miR-146a* in the control individuals and in pre-operative OSCC patients. The *miR-146a* levels in the pre-operative plasma of the patients is significantly higher than that the levels in the control individuals. (E) The before and after plot illustrates the pre-operative to post-operative plasma changes in *miR-146a* for each patient. *miR-146a* can be seen to significantly decrease post-operatively. Horizontal bar, median value. +, mean value. **, *p*<0.01; ***, *p*<0.001. *t*-test. (B, D and F) ROC analysis of A, C and E, respectively, in order to evaluate the separating power when using the -△Ct of *miR-146a*.

An analysis of 60 LCM-retrieved samples (Table S1) showed an increase in *miR-146a* expression in 51 (85%) of the microdissected OSCC tumor cell samples relative to the NCMT samples with mean -△Ct values of 2.59 and 0.04, respectively (Fig. 1A). ROC analysis indicated that the -△Ct of *miR-146a* had a sensitivity and accuracy of 0.72 when separating NCMT and OSCC (Fig. 1B). To examine the feasibility of using the plasma level of *miR-146a* as a diagnostic marker, pre-operative and post-operative plasma were collected from 51 available patients and also sampled from 12 healthy controls. qRT-PCR analysis indicated that the OSCC patients had significantly higher levels of *miR-146a* in their pre-operative plasma than the controls (Fig. 1C) and that this measurement had a sensitivity and accuracy of >0.80 (Fig. 1D) when separating the two groups. When the 36 patients with plasma samples available for both pre-surgical and post-surgical comparison, 81% (29/36) of the patients showed a decline in plasma *miR-146a* after tumor resection to varying degrees (Fig. 1E) and the differences were statistically significant. Analysis showed an accuracy of 0.73 when separating pre-operative and post-operative plasma from OSCC patients using *miR-146a* levels (Fig. 1F). Cases that showed a decline in plasma *miR-146a* after surgery exhibited a trend towards a better survival rate, but the difference was not statistically significant (Table S4).

### Exogenous miR-146a Expression Increases the in vitro Oncogenicity of OSCC Cells

**Figure 2:**
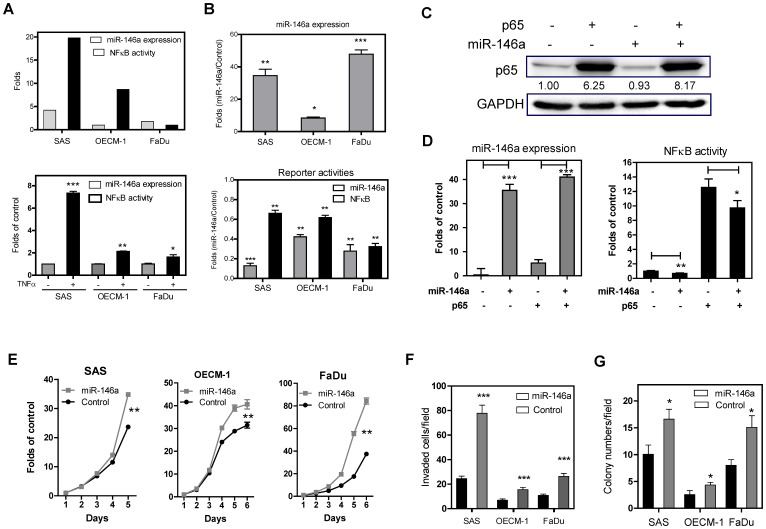
Exogenous *miR-146a* expression enhances OSCC oncogenicity. (A) Upper, The OSCC cells have various endogenous levels of *miR-146a* expression (the level of OECM-1 cells serves as baseline) and of NFκB activity (the level of FaDu cells serves as baseline). Lower, TNFα treatment up-regulates *miR-146a* expression in OSCC cells. (B) The OSCC cell subclones show an increase in *miR-146a* expression (Upper), an increase in *miR-146a* activity and a decrease in NFκB activity (Lower). (C, D) Exogenous p65 expression in SAS cell subclones. (C) Western blot analysis showing exogenous p65 expression after transfection. (D) *miR-146a* expression (Lt) and NFκB activity (Rt). The analysis shows that *miR-146a* expression only slightly down-regulates endogenous or exogenous NFκB activity. The fold changes in (D) are determined by normalization against the control cells transfected with empty vector. (E - G) Proliferation assay, invasion assay and AIG analysis performed on the OSCC cell subclones. Exogenous *miR-146a* expression in OSCC cells increases these oncogenic phenotypes to different extents. The folds in (E) are defined by normalization against the cell numbers at different time points relative to the numbers of cell seeded. Data are shown as mean ± SE from at least triplicate analyses. (C) is a representative picture of three individual experiments and the numbers are the normalized values. *, *p*<0.05; **, *p*<0.01; ***, *p*<0.001; Mann-Whitney test or Two-Way ANOVA test.

SAS, OECM-1 and FaDu cells have different levels of endogenous *miR-146a* expression (Fig. 2A, Upper), and treatment with TNFα is able to up-regulate *miR-146a* to different degrees (Fig. 2A, Lower). SAS cells have the highest level of endogenous *miR-146a* expression and show the strongest response to TNFα treatment relative to other cells. The above cells were infected with lentiviral virus carrying *miR-146a* and with lentivirus control vector. Stable cell subclones were achieved by sorting for green fluorescence. qRT-PCR analysis demonstrated a high level of exogenous *miR-146a* expression in sorted cell subclones (Fig. 2B, Upper). There was also a decreased β-galactosidase activity levels in these stable cells after transfection with the *miR-146a* reporter plasmid (Fig. 2B, Lower), suggesting the presence of exogenous *miR-146* activity. These OSCC cell lines exhibited variable levels of endogenous NFκB activity (Fig. 2A, Upper). Exogenous *miR-146a* expression was found to slightly decrease NFκB activity, but this was less than the reduction in *miR-146a* activity (Fig. 2B, Lower). To further validate this relationship, the NFκB p65 subunit was transfected into a SAS cell subclone (Fig. 2C). Exogenous p65 expression increased *miR-146a* expression by about five folds (Fig. 2D, Lt), but this increase in *miR-146a* expression only slightly suppressed endogenous and exogenous NFκB activity (Fig. 2D, Rt). OSCC cell subclones with exogenous *miR-146a* expression were found to have significantly higher proliferation, invasion and AIG values relative to the controls (Fig. 2E - G, respectively). The increase in invasion was very obvious across all cells. However, various signaling elements in these subclones, namely AKT, ERK, JNK and p38, were found not to be consistently activated following exogenous *miR-146a* expression (Fig. S1).

### Exogenous miR-146a Expression Increases the Tumorigenicity and Metastasis of OSCC Cells

**Figure 3:**
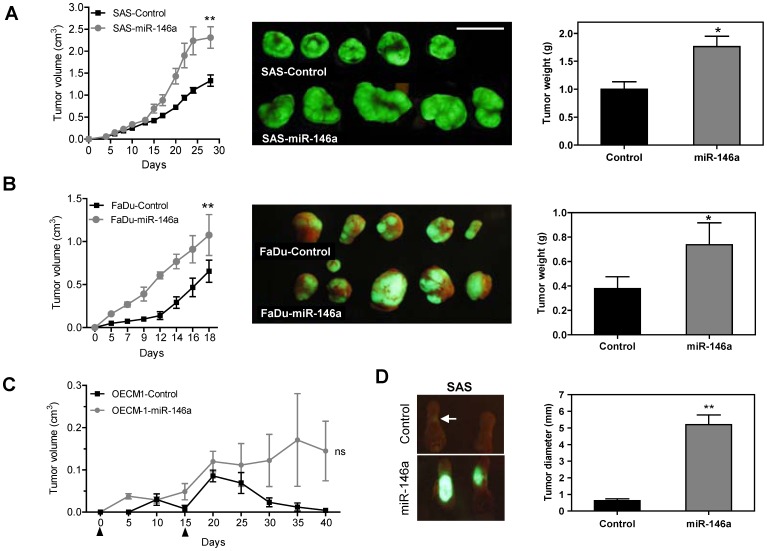
Exogenous *miR-146a* expression enhances the tumorigenesis of OSCC. (A–C) Subcutaneous tumorigenesis of SAS, FaDu and OECM-1 cells, respectively, in an athymic mouse model. (A, B) Lt, the growth curves of the xenografts. Middle, fluorescent photographs of the resected tumors after sacrifice. Rt, quantification of the tumor weight. (C) The growth curve of the OECM-1 xenografts. Arrowheads indicate the time points for tumor cell inoculation. (D) Orthotopic tongue tumorigenesis of SAS cell subclones. Lt, fluorescent tumors caused by the SAS cell subclones with exogenous *miR-146a* expression are present in the tongue of the test animals, but not in the control animals. An arrow indicates the barely visible fluorescent mass in a control mouse. Rt, quantification of the tumor size. Data shown are the means ± SE from at least six mice. *ns*, not significant; *, *p*<0.05; **, *p*<0.01; Mann-Whitney test or Two-Way ANOVA test.

**Figure 4:**
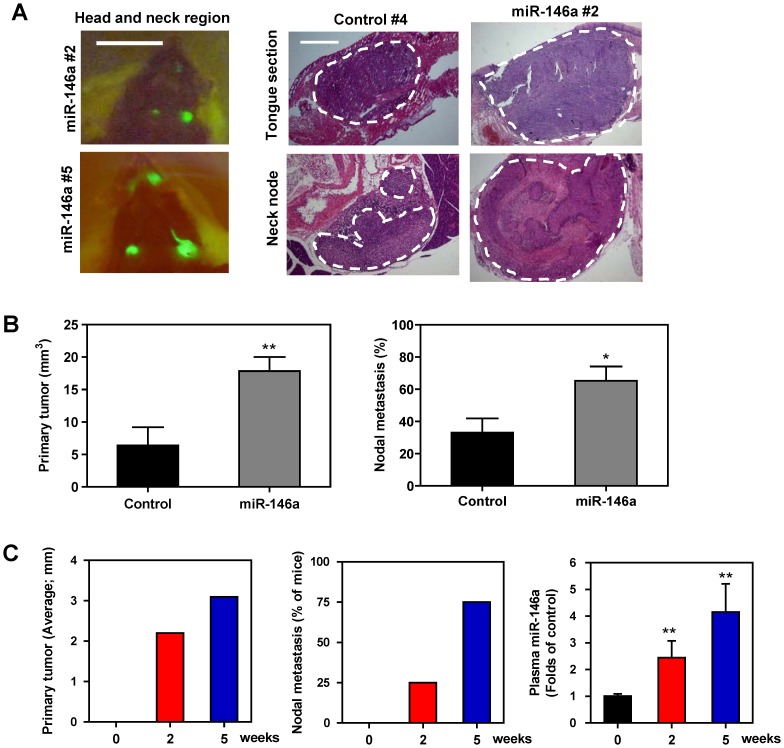
Exogenous *miR-146a* expression enhances the orthotopic tumorigenesis and neck nodal metastasis of SAS cells. (A, B) Orthotopic tongue tumorigenesis and neck nodal metastasis of SAS cell subclones using the NOD-SCID mouse. (A) Lt, fluorescence images of head and neck region. Fluorescent primary tongue tumors and neck metastatic lesions are noted *in vivo* prior to autopsy. Middle panels, representative histopathological sections of the tongue and neck nodes. Bars, 1 cm or 1 mm (in Rt panels). The dotted lines delineate the lesions. Magnification of the histopathological sections, x25. (B) Quantification. Lt, the volume of primary tumors; Rt, the incidence of nodal metastasis. Exogenous *miR-146a* expression is significantly associated with increased burden of primary tumor burden and neck metastasis of mice. Data shown are the means ± SE from at least six mice. *ns*, not significant; *, *p*<0.05; **, *p*<0.01; Mann-Whitney test. (C) Quantification of the diameter of orthotopic tumors (Lt); the percentage of mice exhibiting visible or palpable neck masses (Middle); the plasma *miR-146a* (Rt) at different time points following the orthotopic injection of the SAS cell subclone with exogenous *miR-146a* expression. Progressive increase of plasma *miR-146a* that occurs in parallel with tumor growth and neck metastasis is noted. Data shown are the means or means ± SE from four mice. **, *p*<0.01; Wilcoxon’s test.

SAS cells and FaDu cell subclones with exogenous *miR-146a* expression showed significantly greater subcutaneous xenograft tumor growth compared to control cells (Fig. 3A and Fig. 3B, respectively). OECM-1 is a non-tumorigenic OSCC cell line. When OECM-1 tumor cells were inoculated twice subcutaneously (on days 1 and 15), the OECM-1 cell subclone with exogenous *miR-146a* expression gave rise to small xenograft tumors, while the controls did not exhibit any tumorigenesis until about the 6^th^ week (Fig. 3C). Xenografts of a SAS cell subclone with exogenous *miR-146a* expression grew rapidly on lateral tongue in athymic mice and caused the mice to reach a severely diseased status that require sacrifice at two to three weeks after inoculation (Fig. 3D). However, the growth of the control cells was limited. Thus, because there seems to be a great discrepancy in growth potential between the various cell types used in this model system, a NOD-SCID orthotopic model was adopted to further evaluate primary tumor induction and metastasis [46]. The SAS cell subclone with exogenous *miR-146a* expression exhibited significantly faster orthotopic growth than the controls over five weeks. Moreover, regional metastasis was also clearly observed in the mice injected with the exogenous *miR-146a* expression SAS cell subclone by fluorescence image analysis (Fig. 4A, Lt). A histopathological examination revealed the orthotopic growth of tumors in tongue and the presence of metastatic lesions in the neck lymph nodes (Fig. 4A, Rt). Representative histopathological analysis of the primary tumors and neck metastatic nodes are presented in Fig. S2. Quantification indicated that the SAS cell subclone with exogenous *miR-146a* expression exhibited faster tumor growth (Fig. 4B, Lt) and a higher incidence for neck nodal metastasis (Fig. 4B, RT). The size of the orthotopic tumors and the incidence of neck masses in mice progressively increased from before inoculation, to 2-week after inoculation and then to 5-week after inoculation of SAS cell subclone with exogenous *miR-146a* expression (Fig. 4C, Lt and Middle). Plasma *miR-146a* levels also increased and following the severity of tumor progression in mice (Fig. 4C, Rt).

**Figure 5:**
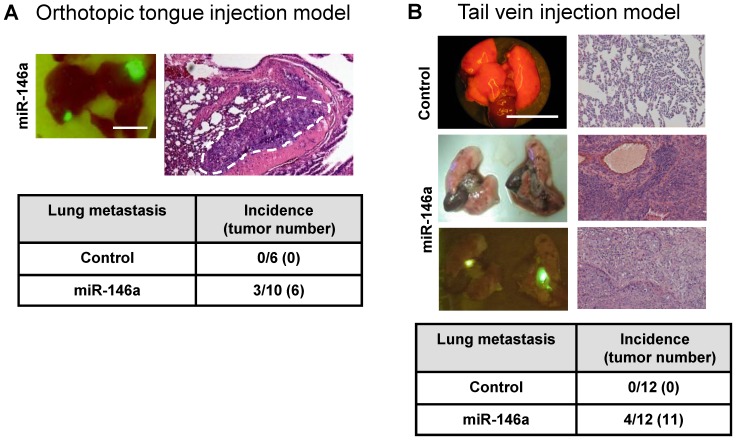
Association between exogenous *miR-146a* expression and lung metastasis in SAS cells. (A) Orthotopic tongue injection and lung metastasis model using the NOD-SCID mouse. Upper, a fluorescent image showing the distal metastatic lesions in the lungs (Lt) and in a histopathological section (Rt). The dotted line delineates the lesion. Lower, a summary of the lung metastasis. (B) Tail vein injection metastasis model. Upper Lt, representative gross and fluorescence images of lungs; Rt, representative histopathological sections of lung tissues showing un-involved lung (Upper picture) and the metastatic carcinomas (Middle and Lower pictures). Lower, a summary of the lung metastasis. Bars, 1 cm. Magnification of the histopathological sections, x100. Numbers in parentheses are tumor numbers. SAS cell subclone with exogenous *miR-146a* expression has a greater metastasis potential in relation to the controls.

Imaging and histopathological analysis revealed that three NOD-SCID mice inoculated with the SAS cell subclone with exogenous *miR-146a* expression also showed lung metastasis (Fig. 5A). However, no lung metastasis was noted in control mice (Fig. 5A, Lower). As the differential neck metastasis could still be a result of the differential primary tumor burden between the mice, the tail vein injection model was employed to investigate further the metastasis potential of the various cell lines. Seven weeks after injection of the SAS subclone cells, the athymic mice were sacrificed and an autopsy was performed to detect the presence of fluorescent foci in lungs, liver and abdomen. Representative fluorescent foci in lungs are shown in Fig. 5B. Histopathological examination confirmed that tumors were present in the lungs of 33% (4/12) of the mice injected with the SAS cell subclone with exogenous *miR-146a* expression, while no mouse injected with the control cells exhibited metastasis in any internal organ including the lungs (Fig. 5B, Lower). Thus it can be concluded that, using a variety of mouse models, increased *miR-146a* expression enhances both OSCC cell primary tumorigenesis and OSCC cell tumor metastasis.

### Blockage of miR-146a Expression Results in an Inhibition of OSCC Oncogenicity

**Figure 6:**
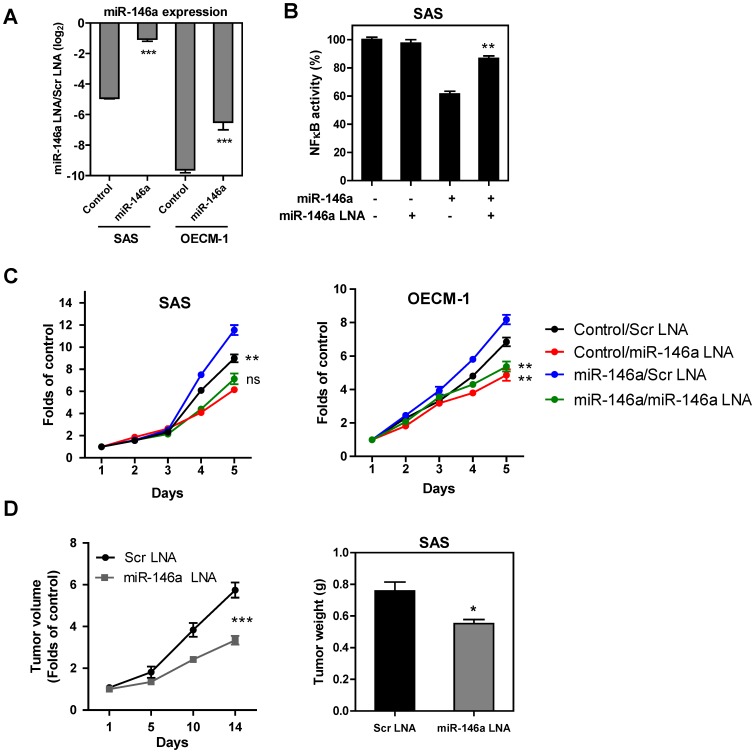
*miR-146a* LNA treatment inhibits OSCC oncogenicity. (A) qRT-PCR analysis. After treatment with *miR-146a* LNA, endogenous and exogenous *miR-146a* expression is reduced. (B) The *miR-146a* LNA treatment results in an increase in NFκB activity, which had been down-regulated by exogenous expression of *miR-146a* in the SAS cells. (C) *miR-146a* LNA treatment decreases the growth of SAS cells (Lt) and OECM-1 cells (Rt) with or without exogenous *miR-146a* expression. The folds are determined by normalization of cell numbers at different time points to the numbers of cell seeded. (D) *miR-146a* LNA represses the tumorigenesis of SAS cells. Lt, growth curve of xenografts. The folds are determined by normalization of tumor volume at different time points to the tumor volume at the time point receiving the injection of *miR-146a* LNA. Rt, quantification of the weight of resected tumors. Data shown are the means ± SE from at least triplicate analysis or five mice. Scr, scramble. *ns*, not significant; *, *p*<0.05; **, *p*<0.01; ***, *p*<0.001; Mann-Whitney test or Two-Way ANOVA test.

Transfection with 100 nM *miR-146a* LNA after 36 h (dosage pre-optimized in pilot tests) decreased *miR-146a* expression in both the control SAS cells, control OECM-1 cells and the cell subclones with exogenous *miR-146a* expression (Fig. 6A). The blockage of *miR-146a* expression slightly up-regulated NFκB activity in the SAS cells (Fig. 6B). This blockage also decreased the proliferation rate of the OSCC cells (Fig. 6C). To test the preclinical efficacy of *miR-146a* blockage in terms of abrogation of OSCC tumor growth, *miR-146a* LNA and scramble LNA were complexed with atelocollagen and injected into the periphery of xenografic tumors induced by the parental SAS in athymic mice [3]. After two weeks, a significant decrease in tumor size of about 37% was found in the tumors treated with *miR-146a* LNA relative to the controls (Fig. 6D).

### miR-146a is Able to Down-regulate IRAK1, TRAF6 and NUMB Expression in OSCC Cells

**Figure 7:**
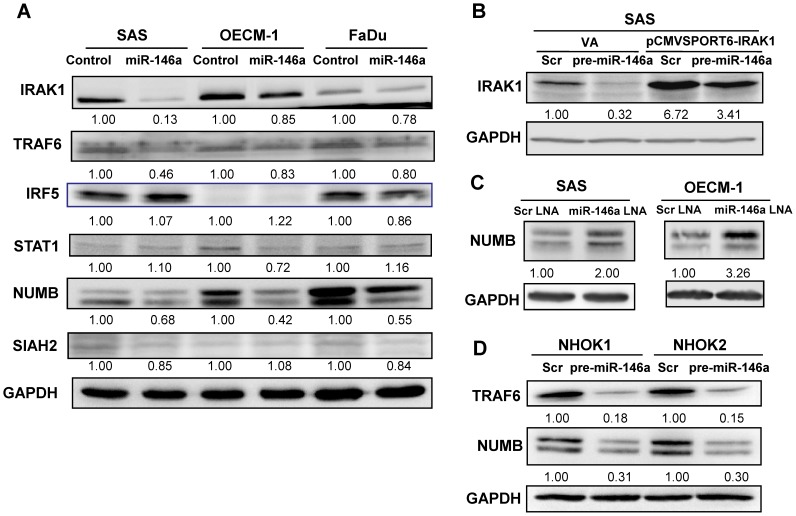
*miR-146a* expression modulates IRAK1, TRAF6 and NUMB expression. Western blot analysis. (A) There is a decrease in IRAK1, TRAF6 and NUMB expression in OSCC cell subclones with exogenous *miR-146a* expression. The decrease in IRAK1 and TRAF6 is particularly prominent in SAS cells. The decrease in NUMB is more prominent than that of IRAK1 and TRAF6 in the three OSCC cell lines. No consistent down-regulation of IRF5, STAT1 and SIAH2 can be seen. (B) Transfection of pre-*miR-146a* mimic and pCMVSPORT6-IRAK1 plasmid in SAS cells. The transfection of pCMVSPORT6-IRAK1 plasmid gives rise to exogenous IRAK1 expression. *miR-146a* expression down-regulates both endogenous and exogenous IRAK1 expression. (C) *miR-146a* LNA treatment increases NUMB expression in SAS and OECM-1 cells. (D) Transfection of the pre-*miR-146a* mimic decreases TRAF6 and NUMB expression in two distinct normal human oral keratinocyte cell lines. Scr, scramble, VA, vector alone. The pictures are representative pictures of two individual experiments. The numbers below the figures are the normalized values.

*miR-146a* has been reported to target the NFκB regulators IRAK1 and TRAF6, as well as the IFN regulators IRF5 and STAT1. *miR-146a* has also been shown to inhibit NUMB and to target Smad4. In addition, *in silico* modeling has predicted that the tumor suppressors SIAH2 and ST7L are also potential targets of *miR-146a*. Western blot analysis showed that there was an unequivocal down-regulation of IRAK1, TRAF6 and NUMB in all OSCC cell subclones with exogenous *miR-146a* expression (Fig. 7A). Protein expression of IRF5, STAT1 and SIAH2, and the mRNA expression of *ST7L* were not affected by *miR-146a* in OSCC cells (Fig. 7A and Fig. S3). Furthermore, reporter assays were also able to exclude SIAH2 and ST7L as targets of *miR-146a* (Fig. S3). In addition, *miR-146a* did not seem to down-regulate Smad4 in OSCC cells or have a consistent effect on the regulation of p-Smad2 or p-Smad3 (Fig. S4). After transfection with the pre-*miR-146a* mimic, the endogenous IRAK1 of SAS cells was down-regulated. Exogenous IRAK1 expression, which was mediated by transfection with pCMVSPORT6-IRAK1 plasmid, was also down-regulated by *miR-146a* expression due to the presence of appropriate 3′UTR sequences in this plasmid clone (Fig. 7B). After treatment with *miR-146a* LNA, NUMB expression in OSCC cells was increased (Fig. 7C). The down-regulation of NUMB and TRAF6 by increased *miR-146a* expression was also found to occur in normal oral keratinocytes (Fig. 7D).

### Knockdown of IRAK1 and TRAF6 Enhances the Oncogenicity of SAS Cells

**Figure 8:**
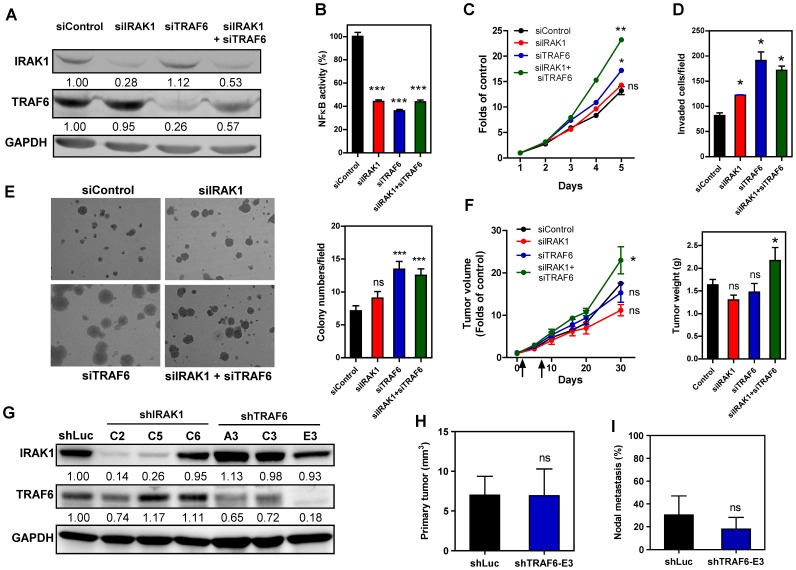
Knockdown of IRAK1 and TRAF6 increases SAS cell oncogenicity. (A–F) Transient knockdown of gene expression using small interference oligonucleotides. (G–I) Stable knockdown of gene expression using shRNA constructs. (A, G) Western blot analysis of IRAK1 and TRAF6 to reveal the knockdown effect. (A) A decrease in IRAK1 and TRAF6 expression is found following treatment with siIRAK1 and/or siTRAF6 oligonucleotides. (B) NFκB activity assay. This shows that NFκB activity is decreased following knockdown of IRAK1 and/or TRAF6. (C – E) Knockdown of IRAK1 and TRAF6 increases the proliferation, invasion and AIG of SAS cells. (E, Lt), inverted microscopy images of colonies; (E, Rt) quantification of colonies. (F) Knockdown of IRAK1 and/or TRAF6 for subcutaneous tumorigenesis. Lt, growth curve. Combined treatment with both siIRAK1 and siTRAF6 significantly increases the xenografic growth of SAS cells. Rt, quantification of the weight of the resected tumors. Arrows indicate time points for oligonucleotide injection. (G) Various shRNA constructs mediate different efficiencies in knocking down IRAK1 or TRAF6 expression in SAS cell subclones. shLuc, control subclone. shTRAF6-E3 subclone and control subclone are subjected to orthotopic tumorigenesis and nodal metastasis assays. (H, I) Volume of orthotopic tumor and rate of nodal metastasis, respectively. Numbers in (A, G) are normalized values. Data shown are the means ± SE from at least triplicate analysis or six mice. *ns*, not significant; *, *p*<0.05; **, *p*<0.01; ***, *p*<0.001; Mann-Whitney test or Two-Way ANOVA test.

To determine whether the expression levels of IRAK1 and TRAF6 are associated with oncogenicity in SAS cells, transfection with siIRAK1, siTRAF6 or both oligonucleotides was carried out to knock down their expression (Fig. 8A). NFκB activity was found to be decreased after knockdown of IRAK1 and TRAF6 (Fig. 8B). Interestingly, an increase in the proliferation, invasion and AIG levels of SAS cells was also found after the knockdown of IRAK1, TRAF6 or both (Fig. 8C–E). The transient effect of siTRAF6 in terms of enhancing the *in vitro* oncogenic phenotype seemed to be greater than the effects of siIRAK1, despite that the fact that they showed similar levels of knockdown and have a similar effect when modulating NFκB activity. Next, these two oligonucleotides were complexed with atelocollagen and injected into the periphery of SAS xenografts on days 1 and 7. This combined treatment with both siIRAK1 and siTRAF6 was found to significantly increase the growth of SAS xenografts by about 30% by the end of the experimental period (Fig. 8F). To validate the effect of IRAK1 and TRAF6 for neck metastasis, establishment of shIRAK1 and shTRAF6 subclones was carried out using SAS cells. The shIRAK1 subclones seemed to acquire the disturbance in TRAF6 expression, they were excluded for further *in vivo* studies. shTRAF6-E3 subclone exhibiting decreased TRAF6 expression and the absence of disruption in IRAK1 expression was used for *in vivo* analysis (Fig. 8G). Pilot tests revealed that orthotopic shTRAF6-E3 xenografts grew faster than shLuc control xenografts (not shown). Thereby, 5-week and 4-week after the orthotopic inoculation of shLuc subclone and shTRAF6-E3 subclone, respectively, were defined the end points for evaluating tumor induction and neck metastasis. Analysis indicated that the tumor volume of shTRAF6-E3 subclone was not different from that of control at the end point (Fig. 8H). Nor the incidence of neck node metastasis in shTRAF6-E3 subclone was different from that of control (Fig. 8I).

### miR-146a Targets both the 3′UTR and the Coding Sequence of the NUMB Gene

**Figure 9:**
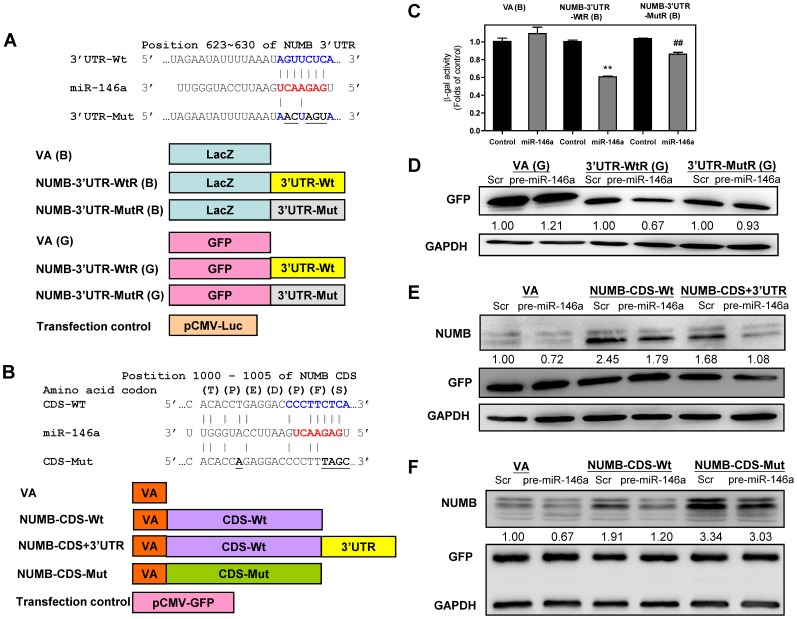
Validation of NUMB as a target for *miR-146a*. (A, B) Schematic diagram of the reporter constructs and expression constructs, respectively. The vertical lines indicate the matching of the sequences. Wt, wild-type; Mut, mutation. CDS, coding sequence; 3′UTR, 3′untranslated region. The characters in parentheses are the one-letter codes for amino acids. The NUMB-CDS-Mut contains mutations affecting five nucleotides, but does not change the amino acid sequence of the protein. (C) Reporter activity assays using LacZ as the reporter gene in SAS cell subclones. Data shown are the means ± SE from triplicate analysis. **, *p*<0.01, comparison with controls; ^##^, *p*<0.01, comparison across WtR(B) and MutR(B); Mann-Whitney test. (D - F) Western blot analysis of the SAS cells transfected with the pre-*miR-146a* mimic, reporter constructs or expression plasmids. (D) GFP expression. SAS cells are transfected with pre-*miR-146a* mimic and GFP reporters. The analysis shows that the wild-type 3′UTR is associated with the repression of GFP expression, while the mutations in the matched sequences reverse the repression. (E, F) NUMB and GFP expression. GFP expression level is used as the transfection control. (E) This shows that the exogenous wild-type NUMB isoform 4 expression in the control cells is decreased in the presence of the 3′UTR, and is further decreased in cells that have exogenous *miR-146a* expression. (F) This shows that the mismatch mutations in the CDS of NUMB reverse the targeting effects of both the endogenous and the exogenous *miR-146a*. Scr, scramble; VA, vector alone. The pictures in (D – F) are representative pictures of two distinctive Western blot analysis. Numbers below gels depict the expression values for GFP normalized against GAPDH (in D) and for NUMB normalized against GFP (in E and F).

A prediction algorithm specified that there was complimentarity between nucleotides 623 to 630 in the NUMB 3′UTR and the seed sequence of *miR-146a*. To validate whether *miR-146a* is able to target NUMB, reporter constructs were generated using the LacZ gene and the GFP gene as reporters; these contained either a wild-type or a mutant 3′UTR of NUMB ligated to the 3′-end of these genes (Fig. 9A). In addition to finding that there was a target site for *miR-146a* in the 3′UTR of NUMB, a similar high affinity target site also was predicted to occur between nucleotides 1000 to 1005 of the NUMB CDS for the seed sequence of *miR-146a*. Constructs carrying the wild-type NUMB (isoform 4) CDS, a wild-type NUMB CDS plus 3′UTR and a mutant NUMB CDS, which had changes to appropriate nucleotides complementary to the *miR-146a* seed sequence but no amino acid replacement, were established (Fig. 9B).

Following the experimental design illustrated in Fig. 9A, the changes in β-galactosidase activity and GFP expression should be modulated by *miR-146a* if there is an appropriate targeting effect. β-galactosidase activity in the wild-type construct was repressed in the SAS cell subclone with exogenous *miR-146a* expression compared to the control cells. Mutation in the target sequence resulted in a significant reversal of β-galactosidase repression (Fig. 9C). Moreover, GFP expression in the wild-type reporter construct was repressed in SAS cells with exogenous *miR-146a* expression compared to the control cells. In agreement with the previous findings, mutation of the target sequence also resulted in a significant reversal of GFP expression (Fig. 9D). These findings suggest that there is binding of *miR-146a* to the target site in 3′UTR of NUMB and that this is able to repress NUMB expression.

According to the experimental design shown in Fig. 9B, with the transfection efficiency normalized using GFP expression, exogenous NUMB expression in control cells from the wild-type NUMB CDS construct was found to be higher than that of the wild-type NUMB CDS plus 3′UTR. When transfection with pre-*miR-146a* mimic was carried out, it was found to result in further down-regulation of the NUMB expression driven by the wild-type NUMB CDS plus 3′UTR construct. These findings further support the presence of a *miR-146a* target in the 3′UTR of NUMB (Fig. 9E). Furthermore, since NUMB expression driven by wild-type NUMB CDS construct was also down-regulated by pre-*miR-146a* mimic transfection. This supports the presence of a potential target site in the NUMB CDS (Fig. 9E). Following the mutation of five nucleotides that should attenuate the affinity between exogenous NUMB and *miR-146a*, it was found that the down-regulation of exogenous NUMB, as mediated by *miR-146a*, underwent partially recovery (Fig. 9F), which substantiates the existence of a *miR-146a* target site in the coding sequence of the NUMB gene.

### NUMB is Able to Mediate OSCC Suppression

**Figure 10:**
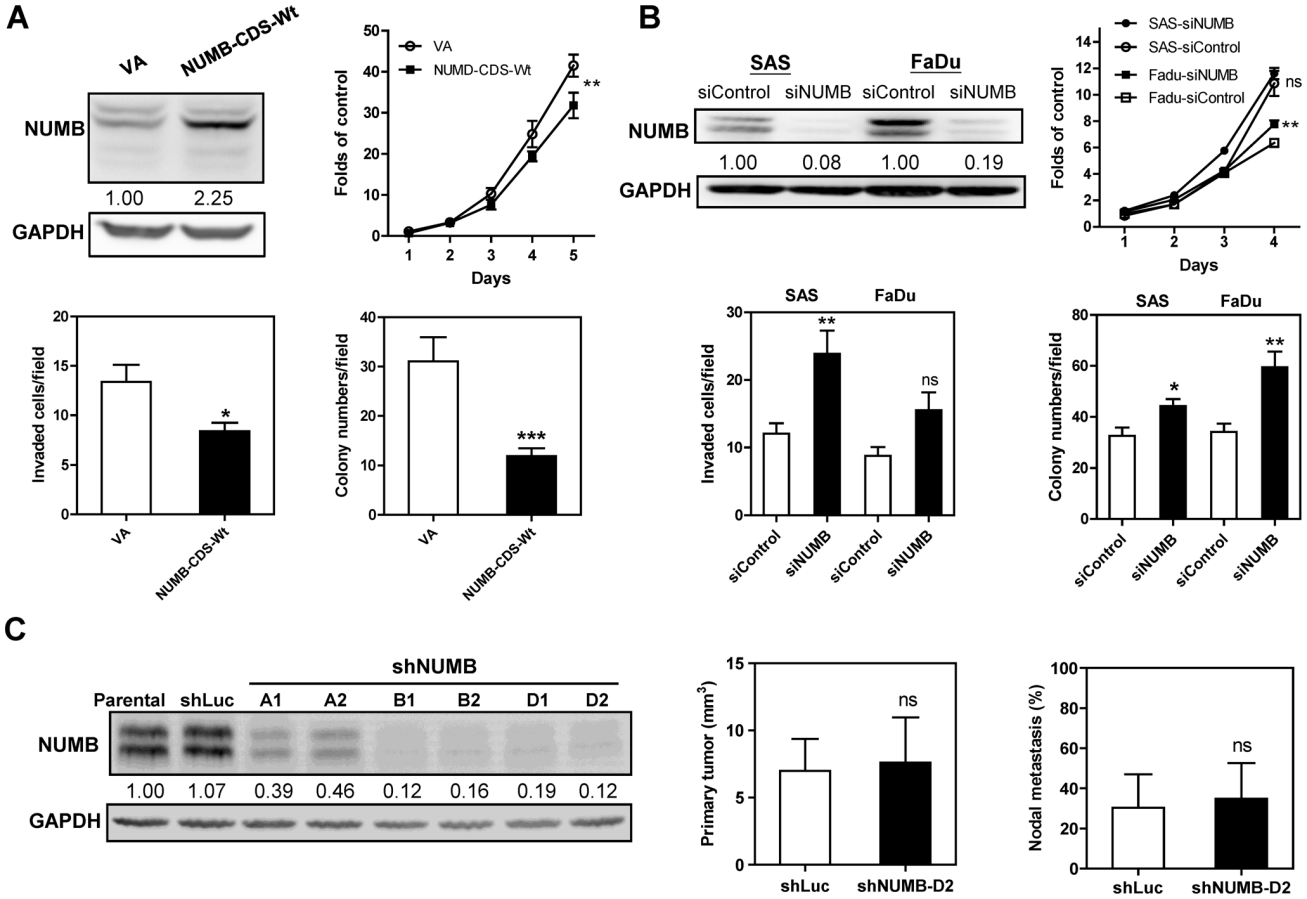
NUMB is an OSCC suppressor. (A) Exogenous expression of NUMB decreases the oncogenicity of SAS cells. The exogenous expression of the NUMB CDS (Upper Lt) decreases the proliferation (Upper Rt), invasion (Lower Lt) and AIG (Lower Rt) of SAS cells. VA, vector alone. (B) Transient knockdown of NUMB using siNUMB increases oncogenicity. Endogenous NUMB expression is drastically decreased after siNUMB treatment in the SAS and FaDu cell lines (Upper Lt). This is associated with a slight increase in the proliferation (Upper Rt), invasion (Lower Lt) and AIG (Lower Rt) of both cell types. (C) Stable knockdown of NUMB expression in SAS cells using shRNA constructs. Lt, different shRNA constructs mediate differential knockdown of NUMB expression in SAS cell subclones comparing to parental SAS cells and shLuc control subclone. shNUMB-D2 subclone is subjected to orthotopic growth and nodal metastasis assays. Middle, volume of orthotopic tumor; Rt, nodal metastasis, respectively. Numbers in the Western blot analysis are normalized values. Data are the means ± SE from at least triplicate analysis or six mice. *ns*, not significant; *, *p*<0.05; **, *p*<0.01; ***, *p*<0.001; Mann-Whitney test or Two-Way ANOVA test.

Exogenous expression of NUMB isoform 4 was found to decrease the proliferation, invasion and AIG levels of SAS cells (Fig. 10A). After treatment with siNUMB, all forms of endogenous NUMB expression in SAS and FaDu cells were remarkably down-regulated. Knockdown of NUMB was found to enhance the growth, invasion and AIG levels of SAS and FaDu cells to different extents (Fig. 10B). The *in vivo* effects of NUMB were analyzed using stable shNUMB subclones derived from SAS cells (Fig. 10C, Lt). Pilot tests disclosed that the xenografic shNUMB subclones grew much faster than control subclone (not shown). The volume of primary orthotopic tumors and the rate of neck nodal metastasis were analyzed 5-week and 3.5-week after the inoculation of shLuc subclone and shNUMB-D2 subclone, respectively. No difference in the tumor volume was noted between shNUMB-D2 subclone and control at the time points for mice sacrifice (Fig. 10C, Middle). On the basis of equal burden in primary tumors, the nodal metastasis rate of shNUMB-D2 subclone was not different from control subclone (Fig. 10C, Rt). Combined knockdown of TRAF6 and NUMB, which simulates *miR-146a* activity, enhanced the *in vitro* oncogenicity of SAS cells, which is then reflected as an increase in growth, invasion and AIG levels of these cells in relation to the controls (Fig. S5).

It was also possible to show that the TNFα-induced down-regulation of NUMB expression was associated with *miR-146a* up-regulation, but that FoxA2 expression in OSCC cells was irrelevant to this event; this is because this event also occurred in cells without FoxA2 expression (Fig. S6A, B). Gli-1, E-cadherin, EGFR and p53 have also been shown to be regulated by NUMB in various types of cells; however, our preliminary assays using both the knockdown and overexpression systems did not support their relevance to *miR-146a* regulation of carcinogenicity in OSCC cells (Fig. S6C and Fig. S7).

**Figure 11:**
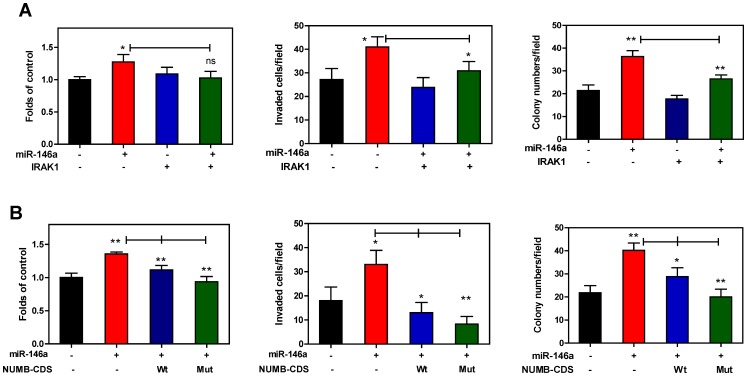
*miR-146a*-mediated oncogenic enhancement is rescued by IRAK1 or NUMB expression in SAS cells. Lt panels, Middle panels and Rt panels, proliferation assay, invasion assay and AIG analysis, respectively. Proliferation assay is performed at the 5^th^ day after cell seeding. (A) IRAK1. Transfection of pre-*miR-146a* mimic and pCMVSPORT6-IRAK1 plasmid were performed. IRAK1 expression is shown in Fig. 7B. The analysis indicates that the invasion and AIG, but not proliferation, induced by *miR-146a* expression are attenuated by exogenous IRAK1 expression. (B) NUMB. Transfection of pre-*miR-146a* mimic and infection with NUMB-CDS-Wt or NUMB-CDS-Mut viruses are performed. Fig. 9F shows that the expression of NUMB in NUMB-CDS-Mut group is higher than that in NUMB-CDS-Wt group. The oncogenic phenotypes that have been enhanced by *miR-146a* expression are significantly attenuated by NUMB expression, especially by NUMB-CDS-Mut. Data shown are means ± SE from triplicate analysis. *ns*, not significant; *, *p*<0.05; **, *p*<0.01; ***, *p*<0.001; Mann-Whitney test.

To address whether *miR-146a* is able to negatively regulate IRAK1 or NUMB in order to enhance OSCC oncogenicity, exogenous IRAK1 or NUMB expression was used to show that the oncogenicity enhancement due to *miR-146a* expression was abrogated by exogenous IRAK1 or NUMB expression in SAS cells. When there was exogenous IRAK1 expression (Fig. 7B), the invasion and AIG enhancements, but not the growth enhancement by *miR-146a* expression, were attenuated (Fig. 11A). In contrast, all oncogenic phenotypes that were enhanced by *miR-146a* expression were abrogated by NUMB to different degrees in SAS cells. In agreement with the fact that the NUMB-CDS-Mut transfection causes higher exogenous NUMB expression than the NUMB-CDS-Wt transfection (Fig. 9F), the NUMB-CDS-Mut transfection produced a more conspicuous attenuation of *miR-146a*-enhanced oncogenicity (Fig. 11B). Thus *miR-146a* seems to modulate oncogenic impact in OSCC by targeting IRAK1 or NUMB.

### Reverse Association between miR-146a Expression and NUMB mRNA Expression in OSCC Tissues

**Figure 12:**
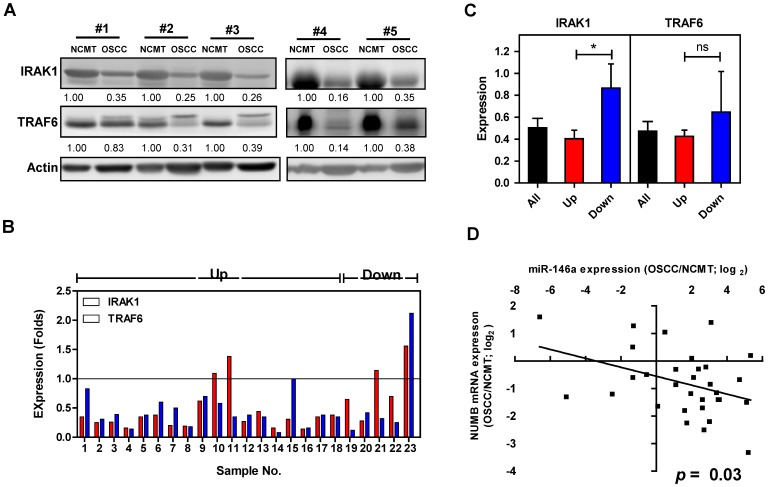
Reverse association between *miR-146a* expression and *NUMB* mRNA expression in OSCC tissues. (A) Western blot analysis for IRAK1 and TRAF6 expression in OSCC and NCMT tissue pairs. Numbers depict the normalized values. (B) Expression of IRAK1 and TRAF6 revealed by the ratio of OSCC/NCMT in 23 OSCC tissue pairs. The vast majority of OSCC exhibits the decreased IRAK1 and TRAF6 expression. Samples 1–18 have *miR-146a* up-regulation, while samples 19–23 have *miR-146a* down-regulation. (C) Expression of IRAK1 and TRAF6 in relation to *miR-146a* expression status. OSCC tissues having *miR-146a* up-regulation exhibit a significantly lower IRAK1 expression. The decrease of TRAF6 expression is not significant in OSCC having *miR-146a* up-regulation. Up, samples having *miR-146a* up-regulation; Down, samples having *miR-146a* down-regulation. Data shown are means ± SE (in C). *ns*, not significant; *, *p*<0.05; Mann-Whitney test. (D) Linear regression analysis of *miR-146a* expression in relation to *NUMB* mRNA expression in 31 OSCC tissue pairs. X-axis: *miR-146a* expression revealed by log_2_ transformation. Y-axis: *NUMB* mRNA expression revealed by log_2_ transformation. Line, regression line. A reverse association between *miR-146a* expression and *NUMB* mRNA expression in OSCC tissues is noted.

To elucidate the correlation of expression levels between *miR-146a* and IRAK1 and *miR-146a* and TRAF6 in OSCC, Western blot analysis was performed using proteins extracted from 23 advanced OSCC tissue pairs. Down-regulation of IRAK1 and TRAF6 expression was found in 19 (83%) and 22 (96%) OSCC samples relative to their respective NCMT samples, respectively (Fig. 12A, B). There were 18 tumors (78%) exhibiting *miR-146a* up-regulation in this tumor subset (Fig. 12B). An association was noted between the *miR-146a* up-regulation (labeled as “Up” in illustration) and lower IRAK1 expression in OSCC tissues (Fig. 12C). However, no association was found between *miR-146a* expression and TRAF6 expression in tissues. Western blot analysis was performed to detect the NUMB protein expression in OSCC tissue specimens. The analysis in pilot tests revealed additional bands admixed with expected NUMB protein bands rendering difficulties in quantitation. Therefore, qRT-PCR analysis was performed to assay *NUMB* mRNA expression in 31 OSCC tissue pairs. Concordant up-regulation of *miR-146a* and down-regulation of *NUMB* was present in 19 (61%) tumors (Fig. 12D). A significant reverse correlation was found between *miR-146a* expression and *NUMB* mRNA expression.

## Discussion

*miR-146* has been found previously to be up-regulated in OSCC [29], [30], [33]. In addition, *miR-146a* expression has been found to be drastically up-regulated in progressive oral precancerous lesions compared to their non-progressive counterparts or compared to normal mucosa [33]. This study confirmed that there is an increase in the level of *miR-146a* in OSCC. HNSCC is known to have high endogenous TNFR activity and high expression of TLRs [10], [11] and in these circumstances the up-regulation of *miR-146a* expression, secondary to NFκB activation, as found in this study, is reasonable. Since the etiological factors of OSCC involve the induction of TNF and the activation of NFκB [41], the association between the oral habits and the *miR-146a* up-regulation in OSCC would be an interesting issue that needs to be addressed. We found that the level of *miR-146a* in tissue has a profound power of prediction with respect to OSCC. The high stability of miRNA in blood enables circulating miRNA to be used as a potential biomarker for malignancies [47]. Our tests also showed that there was a high level of plasma *miR-146a* in OSCC patients compared to the controls. Since plasma *miR-146a* levels in patients were found to decline after the removal of tumors, it seems highly likely that the circulatory *miR-146a* is derived from the tumor tissue [40]. Although a rather strong power of discrimination was noted when using plasma *miR-146a* level to distinguish either cancerous and non-cancerous status or before and after surgery states, this study’s results with respect to the use of plasma *miR-146a* levels as a potential biomarker for distinguishing disease status, or even as a therapeutic target, still needs further validation. This is because changes in *miR-146a* expression may potentially be associated with hematopoietic malignancies and the inflammatory status of subjects [9], [39]. We have identified in a longitudinal animal study that an increase in plasma *miR-146a* parallels the tumor burden of mice that have undergone xenografic transplantation. These findings substantiate strongly the origin of the circulatory *miR-146a* as being from the tumor tissues.

*miR-146a* is up-regulated in HTLV-1-transformed cells [48]. In addition, exogenous *miR-146a* expression is able to increase the cell proliferation of HeLa, NIH3T3 and HCT116 cells [28], [35]. However, expression of *miR-146a* suppresses NFκB activity and this results in a reduction in the metastatic potential of breast cancer cells [36], [37]. The functional roles of *miR-146a* in tumorigenesis therefore remain controversial. This study investigated the oncogenic potential of *miR-146a* in a variety of OSCC cells. It would seem that NFκB is able to promote cell survival and that this activation is common to OSCC [11]. Since the down-regulation of NFκB activity that is induced by high exogenous *miR-146a* expression is not robust, it is possible that *miR-146a* expression is able to modulate other noncanonical effects that overcome the NFκB down-regulation mediated by *miR-146a* expression. The expression of other NFκB family members might also contribute to NFκB activity [5]. One possibility is that there might also be homeostatic regulation, the function of which is to maintain NFκB activity at a stable level in OSCC cells. Although TRAF6 is frequently amplified and acts as an oncogene in non-small cell lung carcinoma [49], the activation of TRAF6 is not crucial to NFκB activation in HNSCC cells [10]. This study showed that *miR-146a* expression is able to down-regulate IRAK1 and TRAF6 in OSCC cells, which is likely to underlie the slight decrease in NFκB activity [7]. In this study, we have provided knockdown evidence demonstrating a strong relationship between TRAF6 expression and OSCC oncogenicity both *in vitro* and *in vivo*. Simultaneous knockdown of IRAK1 and TRF6 further increased the growth of xenografts. It has also been shown previously that TRAF6 is able to modulate the degradation of TGFβRI [50], which could impair the downstream effectors for pathogenesis. Therefore, a potential linkage between *miR-146a* and the phosphorylation of Smad2/3 was investigated. However, our preliminary assays have excluded the possibility that *miR-146a* is able to modulate Smad2/3 activation to a significant degree.

Up to the present, there has been no study that has ever addressed the role of IRAK1in the pathogenesis of HNSCC or OSCC. There is also a lack of clues as to the function of IRAK1 during NFκB activation in HNSCC cells [11]. Knockdown of IRAK1 significantly increased *in vitro* oncogenicity of OSCC cells. By way of contrast, exogenous IRAK1 expression reversed the increase in invasion and *in vitro* transformation that had been enhanced by *miR-146a*. However, more functional *in vivo* approaches are required that validate the mechanistic insight whereby IRAK1 is able to suppress OSCC. As part of the present study, vigorous efforts have been made to detect IRAK1 and TRAF6 expression by immunohistochemical analysis of specimens from SAS xenografts with exogenous *miR-146a* expression in pilot tests. Nevertheless, no convincing decline in the levels of immunoreactivity has been detected using the antibodies available. Western blotting was performed on tissue specimens from advanced tumors that had sufficient amount of proteins available for analysis. However, we noted that there was more prevalent IRAK1 and TRAF6 down-regulation comparing to *miR-146a* up-regulation in this sample cohort. Although it seems likely that other factors in addition to *miR-146a* may be involved in the down-regulation of IRAK1 and TRAF6 during the progression of OSCC [49], the effects of *miR-146a* expression in modulating IRAK1 down-regulation can be shown in advanced tumor samples. The IFN pathway seems to be important for OSCC pathogenesis, whereas our preliminary analysis has excluded the possibility that *miR-146a* targets IRF5, STAT1, Smad4, SIAH2 and ST7L, which have previously either been identified in other types of cells as targets or have been predicted as targets by *in silico* analysis [12]–[14].

The 3′UTR of NUMB is known to contain a *miR-146a* target site [16]. This study discovered that, in addition to this 3′UTR site, there is a novel *miR-146a* target in the CDS of NUMB. *miR-146a* was able to bring about increased repression of NUMB expression in a construct carrying both the wild-type CDS and wild-type 3′UTR compared to a construct carrying only the wild type CDS. The wild-type CDS construct also show higher repression of NUMB expression than a construct carrying a CDS with a mutant *miR-146a* target site [51]. The NUMB isoform 4 is known to be a suppressor in glioblastoma stem-like cells [25]. Another NUMB isoform, but not the NUMB isoform 4, is highly expressed in cervical carcinoma [52]. By knocking down all prominent NUMB isoforms, and overexpressing NUMB isoform 4, we have demonstrated here that NUMB drives suppressor activity in OSCC cells. In this context, the functions of each of the individual NUMB isoforms need to be further elucidated. It is known that TNFα is able to down-regulate NUMB through FoxA2 [27]. In addition to this, our present study proposes that *miR-146a* up-regulation by TNFα is able to knockdown NUMB by targeting both the 3′UTR and CDS of NUMB and that this action is independent of FoxA2 expression. Although NUMB abolishes expression of the NOTCH oncogene in breast cancers, which results in tumor suppression in breast cancers [24], this cannot be the case in HNSCC since NOTCH acts as tumor suppressor in HNSCC rather than as an oncogene [53], [54]. Evidence shows that the Gli-1 oncogene is frequently activated in HNSCC and that this is associated with metastasis [55]. However, our preliminary analysis was unable to identify a reverse association between NUMB and Gli-1 expression in OSCC cells. Our preliminary studies also have excluded an influence of NUMB on the expression of E-cadherin, EGFR and p53 [25], [26], [56]. It is possible that NUMB is able to activate different suppressors in different types of malignancies; in this context the genes regulated by NUMB in OSCC remain to be elucidated.

NUMB isoform 4 is known to drive *in vitro* suppression to OSCC oncogenicity in this study. The eminent down-regulation of *NUMB* mRNA expression contrasting to *miR-146a* up-regulation in the vast majority of OSCC further substantiates its suppressive roles. Besides, a rescue experiment together with other characterizations further clarified that *miR-146a* is able to modulate oncogenicity by targeting NUMB. The combined knockdown of TRAF6 and NUMB showed that NUMB does not seem to act in an additive or counteractive manner with TRAF6 during tumor suppression; it would suggest that TRAF6 and NUMB are likely to mediate independent cascades causing tumor suppression. Although the present study is able to identify a NFκB-*miR-146a* cascade that is involved in OSCC tumorigenesis; this is done by the concomitant targeting of IRAK1, TRAF6 and NUMB, the roles of such parallel targeting in modulating metastasis need to be further addressed. *miR-146a* is involved in multiple types of diseases and therefore the co-targeting of these genes may be important to the mechanisms underlying progression across a variety of diseases. This study shows that *miR-146a* expression is associated with tumorigenesis and metastasis of OSCC. Despite that it is still unclear how it can mediate metastasis, when a *miR-146a* blocker was administered in a mouse model, the xenografic tumorigenesis of the OSCC cells was significantly attenuated. Such an anti-*miR-146a* strategy has the potential to be applied as an OSCC intervention. A greater understanding of the biological functions of *miR-146a* and a clarification of the involvement of various putative targets in relation to the oncogenic behavior of different OSCC may eventually contribute to new diagnostic methods and new targeted therapeutic interventions.

## Supporting Information

Figure S1 ***miR-146a*** **expression and the activation of AKT and MAPK family members in OSCC cells.** Western blot analysis.
(TIFF)

Figure S2 **Histopathological sections.** (A) Orthotopic tongue tumors caused by the SAS cell subclones (B) Neck metastatic lesions of primary tumors in A. x25, dot lines mark lesions.
(TIFF)

Figure S3 **SIAH2 and ST7L are not targets of** ***miR-146a*** **in OSCC cells.** (A) Upper, prediction of the complimentarity between *miR-146a* and the SIAH2 3′UTR sequence. Lower, reporter activity assay. Increased *miR-146a* expression results in no significant change in the level of SIAH2-R reporter activity in OSCC cells. Data shown are mean ± SE from triplicate analysis. *ns*, not significant; Mann-Whitney test. (B) Upper, prediction of the complimentarity between *miR-146a* and the ST7L 3′UTR sequence. RT-PCR analysis (middle) and reporter activity assay (lower) shows that there is no change in *ST7L* mRNA expression or ST7L-R reporter activity after *miR-146a* expression has been modulated. Data shown are representative results from two individual experiments.
(TIFF)

Figure S4 ***miR-146a*** **expression and the expression of Smad family members in OSCC cells.** Western blot analysis. Data shown are representative results from two independent experiments.
(TIFF)

Figure S5 **Double knockdown of TRAF6 and NUMB increases oncogenicity.** Treatment with siTRAF6 and siNUMB significantly increases the proliferation (A), invasion (B) and AIG (C) of SAS cells. Data are the means ± SE from at least triplicate analysis. **, *p*<0.01; ***, *p*<0.001; Mann-Whitney test or Two-Way ANOVA test.
(TIFF)

Figure S6 **The modulation of NUMB expression by** ***miR-146a*** **is independent of FoxA2 in OSCC cells.** Western blot analysis. (A) OSCC cells exhibit consistent levels of TLR4 and IKKα expression, but only SAS cells have detectable FoxA2 expression. Gli-1 expression in OSCC cells is quite weak. (B) *miR-146a* is able to down-regulate NUMB expression in SAS and OECM-1 cells, which have and do not have FoxA2 expression, respectively. *miR-146a* exerts no marked regulation on Gli-1 expression level in SAS and OECM-1 cells. (C) Knockdown of NUMB expression causes no consistent change in Gli-1 expression in a variety of OSCC cells.
(TIFF)

Figure S7 **NUMB expression is not associated with the expression of E-cadherin, EGFR or p53 in SAS cells.** Western blot analysis. (A) Treatment with pre-*miR-146a* mimic or knockdown of NUMB expression does not affect E-cadherin expression. Exogenous NUMB expression does not affect E-cadherin expression either. (B) Exogenous NUMB expression does not activate EGFR or AKT. (C) pre-*miR-146a* treatment for different time periods (Lt), knockdown of NUMB or exogenous expression of NUMB (Rt) does not cause notable change of p53 expression in SAS cells, which possess wild type p53 activity. Scr, scramble; VA, vector alone.
(TIFF)

Table S1 **Clinicopathological parameters of OSCC.**
(DOCX)

Table S2 **shRNA clones used in this study.**
(DOCX)

Table S3 **Antibodies used in this study.**
(DOCX)

Table S4 **Change of plasma** ***miR-146a*** **after surgery as related to patient’s survival.**
(DOCX)
