# Supplementary figures and images for: miR-146a Enhances the Oncogenicity of Oral Carcinoma by Concomitant Targeting of the IRAK1, TRAF6 and NUMB Genes
Source: PLoS One. 2013 Nov 26;8(11):e79926. doi: 10.1371/journal.pone.0079926 (PMC3841223; doi:10.1371/journal.pone.0079926)

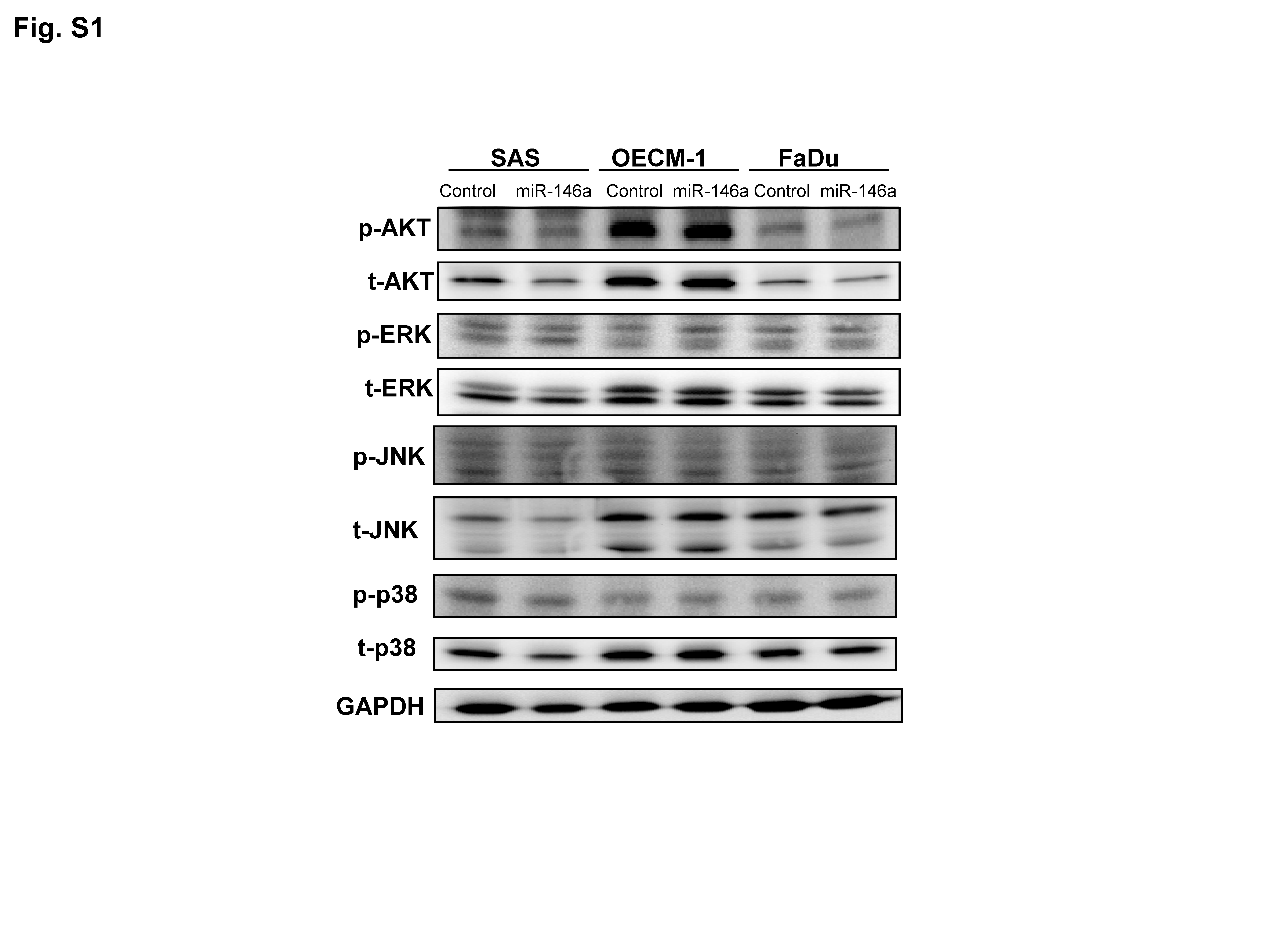

Supplement: Figure S1 — miR-146a expression and the activation of AKT and MAPK family members in OSCC cells. Western blot analysis. (TIFF) [file pone.0079926.s001.tiff]

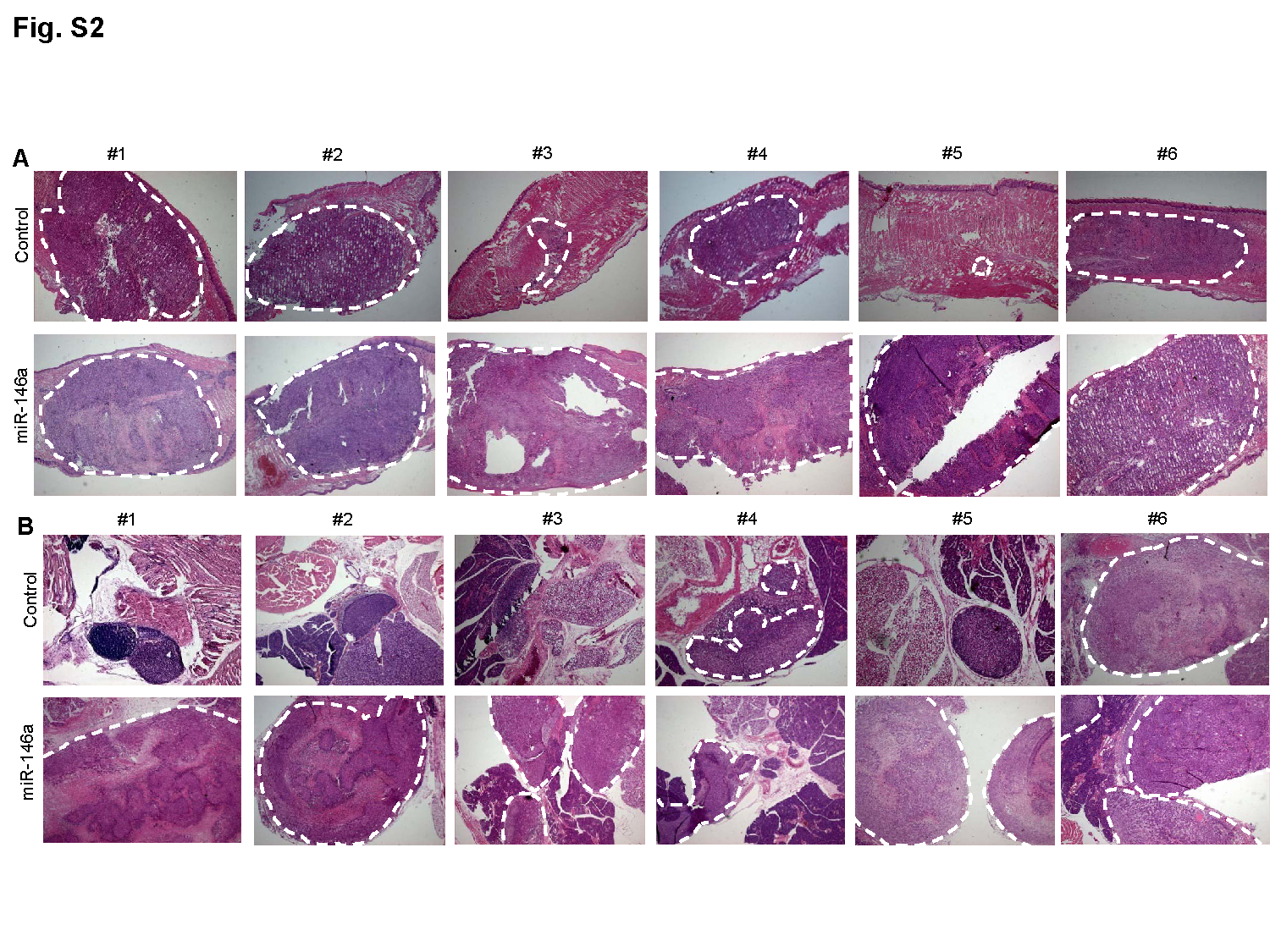

Supplement: Figure S2 — Histopathological sections. (A) Orthotopic tongue tumors caused by the SAS cell subclones (B) Neck metastatic lesions of primary tumors in A. x25, dot lines mark lesions. (TIFF) [file pone.0079926.s002.tiff]

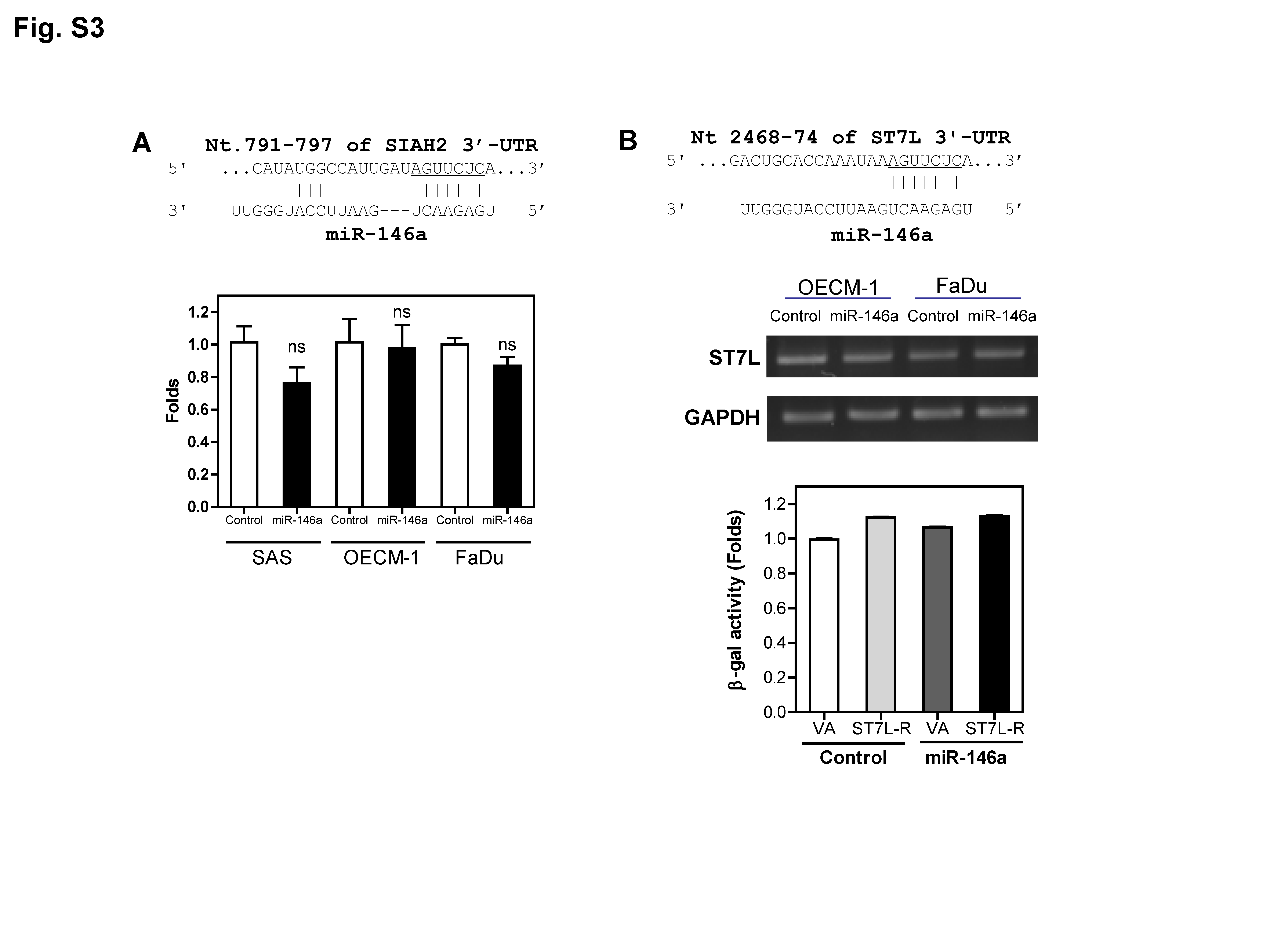

Supplement: Figure S3 — SIAH2 and ST7L are not targets of miR-146a in OSCC cells. (A) Upper, prediction of the complimentarity between miR-146a and the SIAH2 3′UTR sequence. Lower, reporter activity assay. Increased miR-146a expression results in no significant change in the level of SIAH2-R reporter activity in OSCC cells. Data shown are mean ± SE from triplicate analysis. ns, not significant; Mann-Whitney test. (B) Upper, prediction of the complimentarity between miR-146a and the ST7L 3′UTR sequence. RT-PCR analysis (middle) and reporter activity assay (lower) shows that there is no change in ST7L mRNA expression or ST7L-R reporter activity after miR-146a expression has been modulated. Data shown are representative results from two individual experiments. (TIFF) [file pone.0079926.s003.tiff]

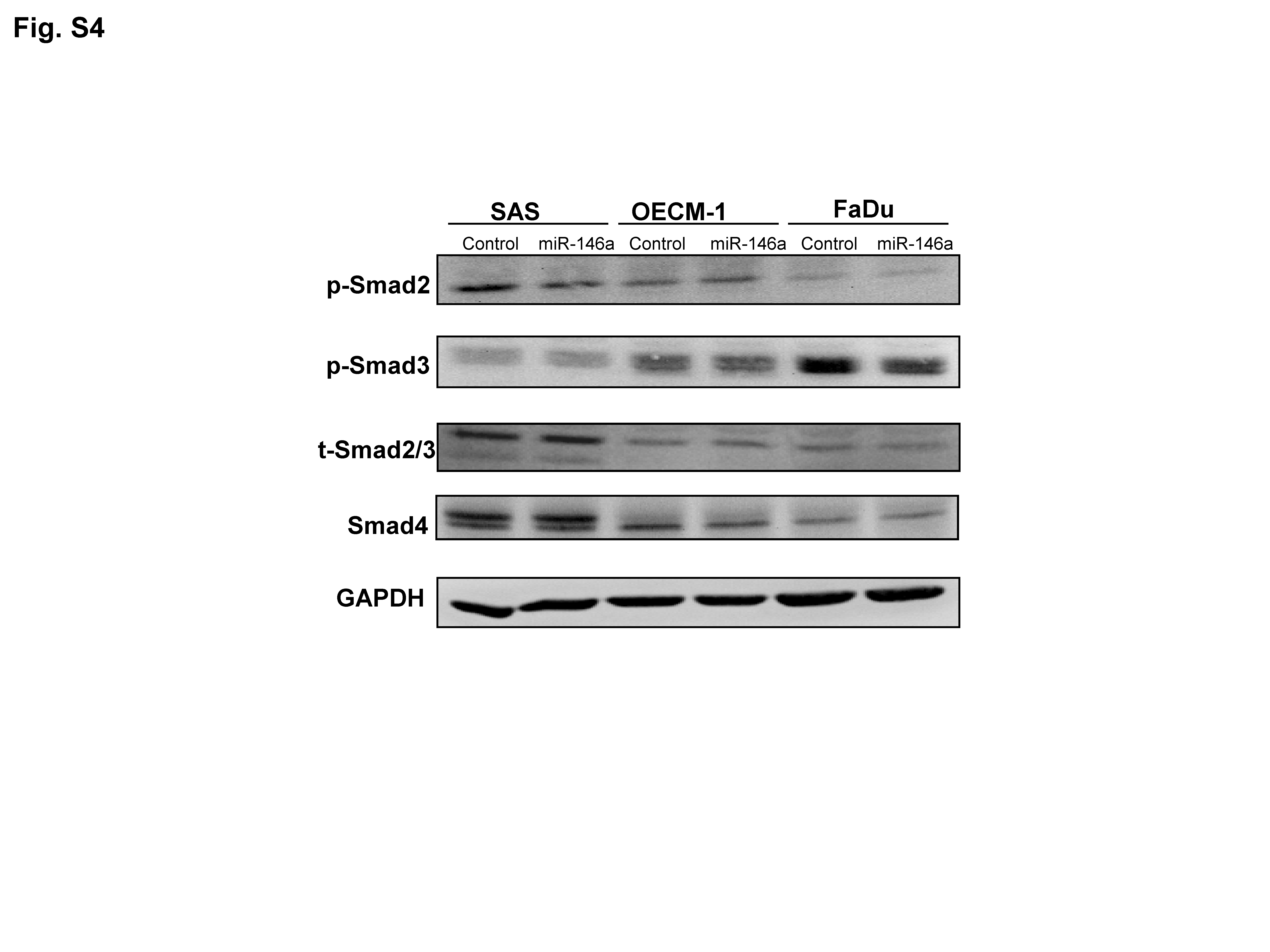

Supplement: Figure S4 — miR-146a expression and the expression of Smad family members in OSCC cells. Western blot analysis. Data shown are representative results from two independent experiments. (TIFF) [file pone.0079926.s004.tiff]

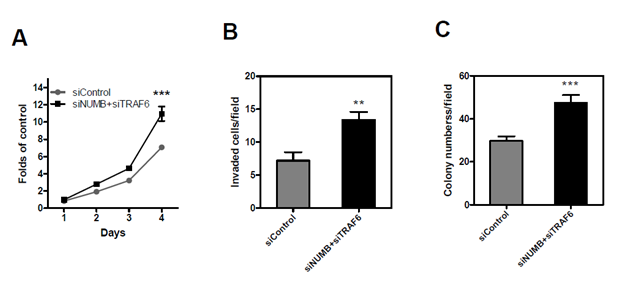

Supplement: Figure S5 — Double knockdown of TRAF6 and NUMB increases oncogenicity. Treatment with siTRAF6 and siNUMB significantly increases the proliferation (A), invasion (B) and AIG (C) of SAS cells. Data are the means ± SE from at least triplicate analysis. **, p<0.01; ***, p<0.001; Mann-Whitney test or Two-Way ANOVA test. (TIFF) [file pone.0079926.s005.tiff]

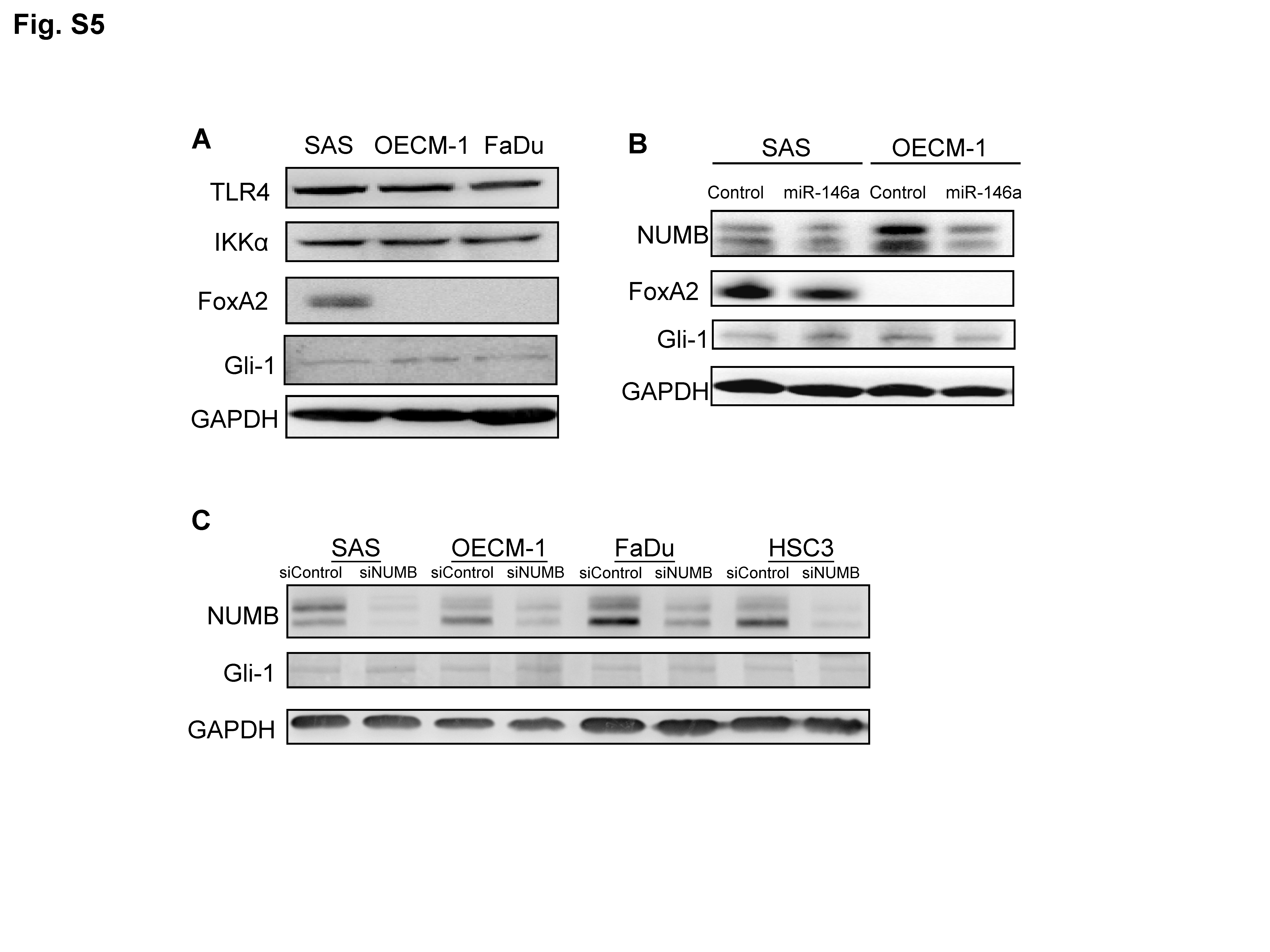

Supplement: Figure S6 — The modulation of NUMB expression by miR-146a is independent of FoxA2 in OSCC cells. Western blot analysis. (A) OSCC cells exhibit consistent levels of TLR4 and IKKα expression, but only SAS cells have detectable FoxA2 expression. Gli-1 expression in OSCC cells is quite weak. (B) miR-146a is able to down-regulate NUMB expression in SAS and OECM-1 cells, which have and do not have FoxA2 expression, respectively. miR-146a exerts no marked regulation on Gli-1 expression level in SAS and OECM-1 cells. (C) Knockdown of NUMB expression causes no consistent change in Gli-1 expression in a variety of OSCC cells. (TIFF) [file pone.0079926.s006.tiff]

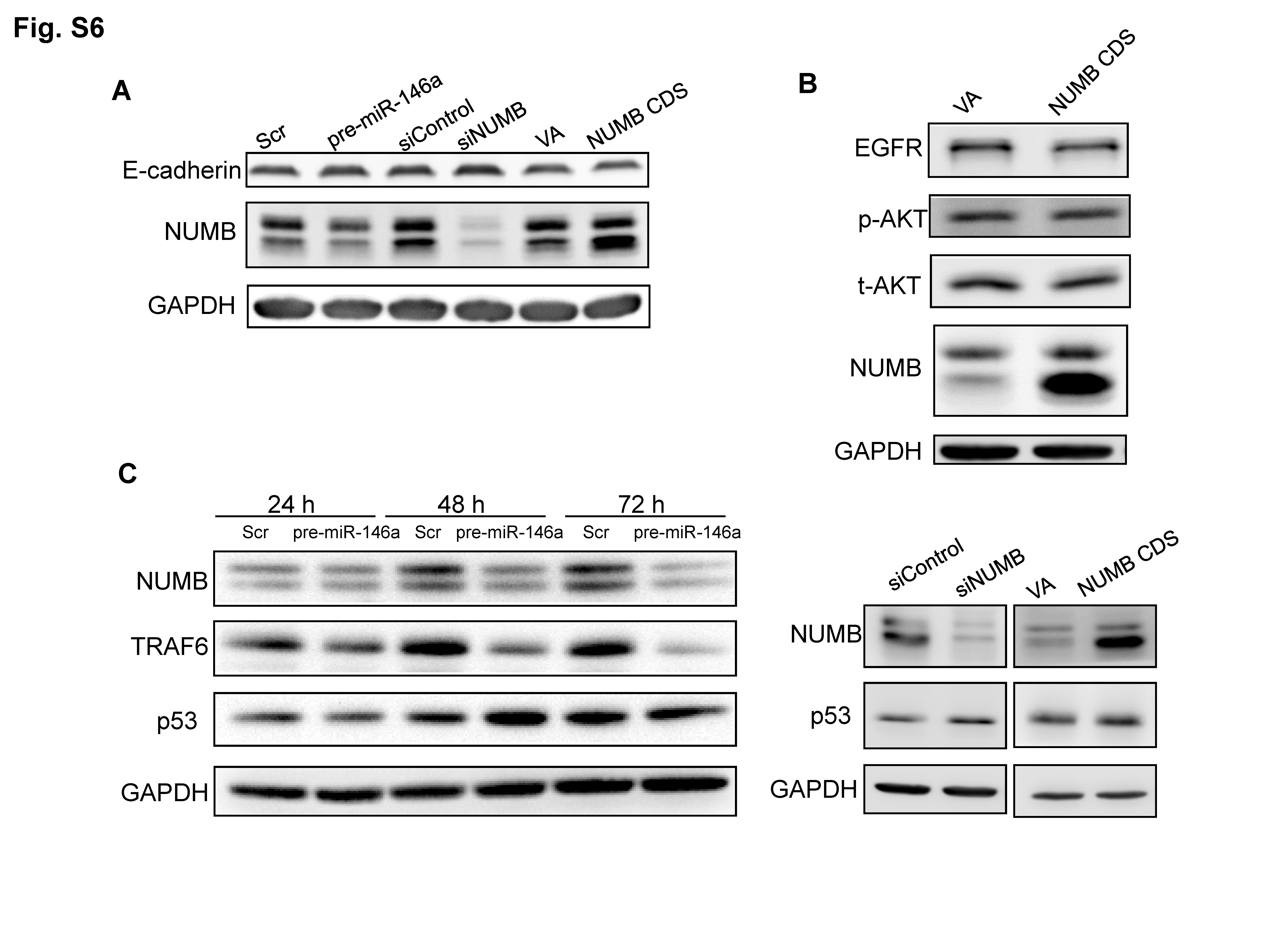

Supplement: Figure S7 — NUMB expression is not associated with the expression of E-cadherin, EGFR or p53 in SAS cells. Western blot analysis. (A) Treatment with pre-miR-146a mimic or knockdown of NUMB expression does not affect E-cadherin expression. Exogenous NUMB expression does not affect E-cadherin expression either. (B) Exogenous NUMB expression does not activate EGFR or AKT. (C) pre-miR-146a treatment for different time periods (Lt), knockdown of NUMB or exogenous expression of NUMB (Rt) does not cause notable change of p53 expression in SAS cells, which possess wild type p53 activity. Scr, scramble; VA, vector alone. (TIFF) [file pone.0079926.s007.tiff]
