# Supplementary material for: miR-146a Enhances the Oncogenicity of Oral Carcinoma by Concomitant Targeting of the IRAK1, TRAF6 and NUMB Genes
Source: PLoS One. 2013 Nov 26;8(11):e79926. doi: 10.1371/journal.pone.0079926 (PMC3841223; doi:10.1371/journal.pone.0079926)
Supplement: Table S1 — Clinicopathological parameters of OSCC. (DOCX) [file pone.0079926.s008.docx]

**Table S1. Clinicopathological parameters of OSCC**

| *n* = | 60 |
| --- | --- |
| Age (Years) | 53.24 ± 9.25 |
| TNM staging |  |
| N0 | 35 |
| N+ | 25 |
| T1-3 | 24 |
| T4 | 36 |
| Stage I-III | 19 |
| Stage IV | 41 |
| Follow-up (Months) | 50.52 ± 24.13 |
| Alive | 35 |
| Dead | 25 |
