# Supplementary material for: miR-146a Enhances the Oncogenicity of Oral Carcinoma by Concomitant Targeting of the IRAK1, TRAF6 and NUMB Genes
Source: PLoS One. 2013 Nov 26;8(11):e79926. doi: 10.1371/journal.pone.0079926 (PMC3841223; doi:10.1371/journal.pone.0079926)
Supplement: Table S2 — shRNA clones used in this study. (DOCX) [file pone.0079926.s009.docx]

**Table S2. shRNA clones used in this study**

| **Clone type** | **ID** | **Target site** | **Target sequence** |
| --- | --- | --- | --- |
| shIRAK1-C2 | TRCN0000000543 | 3UTR | CCCTCCTACCTGCTTACAATT |
| shIRAK1-C5 | TRCN0000000546 | CDS | CATTGTGGACTTTGCTGGCTA |
| shIRAK1-C6 | TRCN0000000547 | CDS | AGGAGTACATCAAGACGGGAA |
| shTRAF6-A3 | TRCN0000007348 | 3UTR | GCCACGGGAAATATGTAATAT |
| shTRAF6-C3 | TRCN0000007350 | CDS | CGAAGAGATAATGGATGCCAA |
| shTRAF6-E3 | TRCN0000007352 | CDS | CCTGGATTCTACACTGGCAAA |
| shNUMB-A | TRCN0000007223 | 3’UTR | CCTTGCAATTAGGCTAAAGAA |
| shNUMB-B | TRCN0000007224 | 3’UTR | GCAATCATTATGGCTATGTAT |
| shNUMB-D | TRCN0000007226 | CDS | GCCATGTAGAAGTTGATGAAT |
