# Supplementary material for: miR-146a Enhances the Oncogenicity of Oral Carcinoma by Concomitant Targeting of the IRAK1, TRAF6 and NUMB Genes
Source: PLoS One. 2013 Nov 26;8(11):e79926. doi: 10.1371/journal.pone.0079926 (PMC3841223; doi:10.1371/journal.pone.0079926)
Supplement: Table S3 — Antibodies used in this study. (DOCX) [file pone.0079926.s010.docx]

**Table S3. Antibodies used in this study**

| Protein | Producer | Dilution |
| --- | --- | --- |
| NUMB | Abcam (Cambridge, MA) | 1:1000 |
| SIAH2 |  | 1:200 |
| E-cadherin | BD Biosciences (San Jose, CA) | 1:2000 |
| p-AKT | Cell Signaling (Boston, MA) | 1:1000 |
| FoxA2 |  | 1:1000 |
| IKKα |  | 1:1000 |
| p-JNK |  | 1:1000 |
| t-JNK |  | 1:1000 |
| p-p38 |  | 1:1000 |
| p-Smad2 |  | 1:1000 |
| p-Smad3 |  | 1:1000 |
| t-Smad2/3 |  | 1:1000 |
| Smad4 |  | 1:1000 |
| GFP | Clonetech Lab (Mountain View, CA) | 1: 5000 |
| TRAF6 | Epitomics (Burlingame, CA) | 1:1000 |
| t-AKT | Santa Cruz Biotech (Santa Cruz, CA) | 1:200 |
| GAPDH |  | 1:10000 |
| Gli-1 |  | 1:200 |
| IRAK1 |  | 1:200 |
| IRF5 |  | 1:200 |
| t-p38 |  | 1:200 |
| p53 |  | 1:200 |
| TLR4 |  | 1:200 |
| Actin | Sigma Aldrich (St Louise, MO)i | 1: 2000 |
| EGFR | Upstate (Billerica, MA) | 1:1000 |
