# Supplementary material for: miR-146a Enhances the Oncogenicity of Oral Carcinoma by Concomitant Targeting of the IRAK1, TRAF6 and NUMB Genes
Source: PLoS One. 2013 Nov 26;8(11):e79926. doi: 10.1371/journal.pone.0079926 (PMC3841223; doi:10.1371/journal.pone.0079926)
Supplement: Table S4 — Change of plasma miR-146a after surgery as related to patient’s survival. (DOCX) [file pone.0079926.s011.docx]

**Table S4. Change of plasma *miR-146a* after surgery as related to patient’s survival**

|  | Alive | Dead | *p* |
| --- | --- | --- | --- |
| Decrease | 19 | 10 | 0.102 |
| Not decrease | 2 | 5 |  |

Fisher’s exact test
